# The impact of size and ontogeny on suction feeding kinematics in the axolotl (*Ambystoma mexicanum*)

**DOI:** 10.1242/bio.061860

**Published:** 2025-07-18

**Authors:** Isabelle Toussaint-Lardé, Vivien Louppe, Frida Sanchez-Garrido, Morgane Taillades, Ryadh Amine, Mark Mandica, Eglantine Heude, Anthony Herrel, Anne-Claire Fabre

**Affiliations:** ^1^ Institute of Ecology and Evolution, Universität Bern, Bern, Switzerland; ^2^ Naturhistorisches Museum Bern, Bern, Switzerland; ^3^ Mécanismes Adaptatifs et Evolution, UMR 7179, Muséum national d'Histoire naturelle CNRS, Paris, France; ^4^ PhyMA, UMR 7221, Muséum national d'Histoire naturelle, CNRS, Paris, France; ^5^ Amphibian Foundation, Atlanta, GA, USA; ^6^ Institut de Génomique Fonctionnelle de Lyon, UMR 5242, École Normale Supérieure de Lyon, France; ^7^ Department of Biology, Evolutionary Morphology of Vertebrates, Ghent University, Ghent, Belgium; ^8^ Department of Biology, University of Antwerp, Wilrijk, Belgium; ^9^ Department of Life Sciences, Natural History Museum, London, UK

**Keywords:** Development, Paedomorphy, Suction feeding, Salamander, Life cycle

## Abstract

Size impacts all aspects of life in animals. Not only does it impact metabolism and physiology, it also affects movements, resulting in different selective pressures on animals of diverse sizes. However, beyond size, differences in developmental stage may also impact movements due to the maturation of the nervous and musculoskeletal systems. Here, we tested the influence of size and ontogeny on suction feeding kinematics in adults, juveniles, and larvae of the axolotl (*Ambystoma mexicanum*) using high-speed video recordings. Our data show that size had an influence on nearly all kinematic variables examined, but kinematics often deviated from the predictions of simple geometric scaling models. Moreover, for both the velocity and acceleration of mouth and hyoid movements, the effect of size differed according to the developmental stage. While overall movements were faster in larger animals, the velocity of movement increased faster with size in adults. This could be explained by the fact that the skull undergoes changes at adulthood due to partial remodeling. Changes in velocity are possibly linked to a shift in diet at adulthood from static to more elusive prey, yet this remains to be tested.

## Teaser

**Summary:** In *Ambystoma mexicanum* larvae, juveniles and adults all use suction feeding. In this research, kinematic analysis reveals shifts in the speed and acceleration of jaw and hyoid movements between immature and adult individuals.

## INTRODUCTION

The size of an organism is of crucial importance as it constrains its structure and, consequently its function (Brown et al., 2000). During ontogeny, animals change in size, which requires appropriate adjustments in behavior or changes to the organism's structure because of the differential scaling between linear dimensions, surface areas and forces, and volume or mass (Schmidt-Nielsen, 1984). Whereas surface areas and forces increase with linear dimensions to the second power, volume and mass increase to the third power resulting in larger animals having relatively lower forces available to move a given structure if they grow geometrically (Schmidt-Nielsen, 1984; Hill, 1950; O'Reilly et al., 1993). Moreover, juveniles and adults often share the same environment, compete for the same resources, and often must escape the same predators (Carrier, 1996). As juveniles are smaller, less developed, and have less experience than adults, they may be at a selective disadvantage (Schmidt-Nielsen, 1984; Carrier, 1996; Herrel and Gibb, 2006). Consequently, differences in growth trajectories can be expected throughout ontogeny.

Theorical models have been developed to predict consequences of changes in size on the musculoskeletal dynamics, such as Hill's geometric similarity model (Hill, 1950). This model assumes isometric scaling of morphology resulting in allometric relationship between the mass of the skeleton and force of the muscles, as the force of a muscle decreases relative to its mass through ontogeny (Hill, 1950; O'Reilly et al., 1993; Robinson and Motta, 2002). Previous scaling studies concern mostly animal locomotion and aquatic feeding in salamanders and bony fish (Otten, 1983; Ashley et al., 1991; O'Reilly et al., 1993; Ashley-Ross, 1994; Reilly, 1995; Richard and Wainwright, 1995; Hernandez and Motta, 1997; Wainwright and Shaw, 1999; Robinson and Motta, 2002). These studies have highlighted contrasting results (Deban and O'Reilly, 2005; Robinson and Motta, 2002). For instance, in spotted salamander larvae (*Ambystoma maculatum*; Hoff et al., 1985) and fire salamander larvae (*Salamandra salamandra;* Reilly, 1995), no changes in aquatic feeding kinematics were observed, while positive correlations in feeding kinematics and growth were observed in toads (O'Reilly et al., 1993) and leopard sharks (Ferry-Graham, 1998). Finally, combinations of different patterns have been observed in fishes such as the woolly sculpin (*Clinocottus analis*; Cook, 1996) and largemouth black bass (*Micropterus salmoides*; Richard and Wainwright, 1995). This means that scaling patterns cannot be generalized from one species to another, which encourages further studies to better understand the impact of size changes.

In addition, for animals undergoing metamorphosis, developmental stage may also impact how movements change during growth. For example, in many fish and salamander species, individuals undergo a partial or complete metamorphosis during which parts of their skull are reshaped (Rose and Reiss, 1993). This may then impact the performance of the feeding system throughout ontogeny.

To try to disentangle the effect of size and developmental stage on feeding kinematics we collected data for an ontogenetic series of the axolotl (*Ambystoma mexicanum*) with individuals of different sizes and developmental stages. *Ambystoma mexicanum* is an instructive model to study the influence of size on feeding kinematics as this species is paedomorphic, meaning that adults retain larval characteristics even when they are sexually mature (notably they still have external gills, gill slits, a tail fin, labial lobes, and they lack eyelids). As a result, at first sight, adults appear externally similar to larvae and juveniles, apart from differences in size. Moreover, they all use suction feeding, which is achieved by creating an antero–posterior expansion wave causing water to flow into the oral cavity (see Figs S1 and S2; Movies 1-3). Due to the density and viscosity of the medium, the prey is sucked into the oral cavity and water is expelled through the gill slits (see Figs S1 and S2; Movies 1-3). We first hypothesized that size would have an influence on the kinematics of prey capture. Indeed, the size of larvae (particularly of their mouth) can have an impact on their capacity to capture prey as it determines the hydrodynamic regime of the flow generated during suction (China and Holzman, 2014). Moreover, the durations of feeding movements in most of the species described to date are positively correlated with changes in head dimensions (Deban and O'Reilly, 2005). We further hypothesized that developmental stage would influence the kinematics of suction as it is intimately linked to the cranial musculoskeletal maturation. Indeed, even if the mandibular, hyoid, branchial, and hypobranchial muscles are functional from larval stage 43 onwards in the axolotl (Ziermann and Diogo, 2013; Ericsson et al., 2004), the ossification of the skull occurs during the morphological growth and differentiation of the limbs (from larval stage 44 to 57; Smirnov and Vassilieva, 2004; Nye et al., 2003). Thus, compared to juveniles and adults, larvae present a mostly cartilaginous skull, which likely is less resistant to muscle forces and so may impact the kinematics of feeding.

## RESULTS

### Do developmental stages differ in size?

See the Materials and Methods for how developmental stages were defined. The ANOVAs revealed that developmental stages differed in size (*F*=77.29, *P*<0.001). Post-hoc tests further revealed that adults are significantly different in size from larvae (*t*=−12.256, *P*<0.001) and juveniles (*t*=−8.83, *P*<0.001) and are on average three times larger (Table S2). However, the juveniles used in this study overlap in size with the larvae, some larvae being even larger than the largest juveniles (Table S2).

### Scaling

See the Materials and Methods for the definitions of isometry and allometry. All the displacement metrics and angles scaled with isometry (Table 2). All the speed and acceleration variables showed positive allometry (Table 2). All the timing and duration variables showed negative allometry compared to the predictions of geometric similarity (Table 2).

### Variables with no significant interaction: the effect of size and development stage

See Table 1 for definitions of the variable abbreviations. The ANCOVAs revealed that the angles ‘MGA’ and ‘MHA’, the timings ‘TMG’, and ‘Thd1’ and the durations ‘DG’ and ‘PCD’ were not significantly impacted neither by size nor by developmental stage. In contrast, size significantly impacted the distances ‘MG’, ‘Mhd1’ and ‘Mhd2’, the times ‘TMHA’, ‘TMhd1’ and ‘TMhd2’, the speeds ‘MSGO’, ‘MShd1down’, ‘MShd2down’, and the acceleration ‘MAhd2down’ (Table 4). In addition, developmental stage significantly impacted the duration variables ‘Dhd1’, ‘Dhd2’ (Table 4). Post-hoc tests showed that the effect of size was no longer present for the time ‘TMHA’ and for ‘MAhd2down’. Similarly, the effect of developmental stage on the duration ‘Dhd1’ was no longer significant in the post-hoc tests (Table S3).

**Table 1.** List of the 25 kinematic variables extracted from the video with associated abbreviations

Type of movements	Kinematic variables	Abbreviations
Jaw movements	Maximum gape distance	MG
	Time to maximum gape distance	TMG
	Maximum gape angle	MGA
	Duration of the gape cycle from opening to closing of the mouth	DG
	Maximum speed of jaw opening	MSGO
	Maximum acceleration of jaw opening	MAGO
	Maximum speed of jaw closing	MSGC
	Maximum acceleration of jaw closing	MAGC
Head movements	Maximum head angle during prey capture	MHA
	Time to the maximum head angle	TMHA
Movements of the anterior part of the hyoid	Time when the anterior point of the hyoid begins to depress	Thd1
	Maximum depression of the anterior part of the hyoid	Mhd1
	Time to maximum depression of the anterior part of the hyoid	TMhd1
	Duration of the cycle of the anterior part of the hyoid	Dhd1
	Maximum speed of the depression of the anterior part of the hyoid	MShd1down
	Maximum speed of the elevation of the anterior part of the hyoid	MShd1up
	Maximum acceleration of the anterior part of the hyoid during depression	MAhd1down
Movements of the posterior part of the hyoid	Time when the posterior point of the hyoid begins to depress	Thd2
	Maximum depression of the posterior part of the hyoid	Mhd2
	Time to maximum depression of the posterior part of the hyoid	TMhd2
	Duration of the cycle of the posterior part of the hyoid	Dhd2
	Maximum speed of depression of the posterior part of the hyoid	MShd2down
	Maximum acceleration of the posterior part of the hyoid during depression	MAhd2down
	Maximum speed of elevation of the posterior part of the hyoid	MShd2up
Total movement	Prey capture duration from the initial opening of the mouth to the return of the hyoid apparatus to its initial position	PCD

Most of the differences in kinematic variables between larvae and adults were explained by size differences (see distances ‘MG’, ‘Mhd1’, ‘Mhd2’, times ‘TMhd1’ and ‘TMhd2’, speeds ‘MSGO’, ‘MShd1down’, ‘MShd2down’; Table S3). Similarly, most of the differences in kinematic variables between juveniles and adults were explained by size differences (see distances ‘MG’, ‘Mhd1’, ‘Mhd2’, duration ‘Dhd2’, speeds ‘MSGO’, ‘MShd1down’; Table S3). Finally, developmental stage played a role in the differences between juveniles and adults for the duration ‘Dhd2’ (Table S3).

### Variables with significant interaction terms (Table 5)

Of the variables with a significant interaction term, all had a significant interaction term when the ANCOVAs were performed on the larval versus adult dataset (group compared: L-A, Table 5). Conversely, there was never any interaction when the ANCOVAs were performed on the larval versus juvenile dataset (group compared: L-J, Table 5). Finally, when ANCOVAs were performed on the juvenile versus adult dataset, the significant interactions concerned the speeds ‘MSGC’, ‘MShd1up’, ‘MShd2up’, and the acceleration ‘MAGC’ (group compared: J-A, Table 5).

When adult axolotls were removed from the dataset (group compared: L-J, Table 5), all kinematic variables were impacted by size except ‘Thd2’ (group compared: L-J, Table 5) and only ‘MShd2up’ was also impacted by developmental stage (group compared: L-J, Table 5). A significant effect of developmental stage on the ‘MAhd1down’ variable can also be noted when the ANCOVA only took juveniles and adults into account (group compared: J-A, Table 5).

### Degree of ossification of the feeding apparatus through ontogeny

The cleared-and-stained specimens revealed that the earlier larval stage (stage 53) shows most of the anterior parts of its feeding structures already ossified (premaxilla, vomer, and the palatine of the palatopterygoid for the cranium and the dentary and the coronoid for the lower jaw). However, the rest of the cranium and the lower jaw is still cartilaginous (Fig. 4). At later larval stages (stage 57), the cranium starts to mineralize, and more structures composing the feeding system are ossified (premaxilla, maxilla, vomer, both palatine and pterygoid of the palatopterygoid, squamosal and part of the quadrate for the cranium and, dentary, coronoid, prearticular and angular for the lower jaw). At the adult stage, the calcification of the cranium is complete with a higher degree of ossification compared to larval stages (Fig. 4). The hyobranchial apparatus and gills remain cartilaginous at all stages (Fig. 4).

## DISCUSSION

### The impact of size on feeding kinematics through development

The comparison of size among the different developmental stages in our dataset showed that adults were significantly larger than larvae and juveniles. In contrast, larvae and juveniles overlapped in size. This means that size is not a good proxy to determine the developmental stage, and also that growth is not isometric in this species. If growth was isometric, juveniles would be bigger in size than the late larvae. It is then not surprising to find that most of the kinematic variables did not scale isometrically relative to the predictions of a Hill model. As growth is not isometric, a Hill model (1950) is likely not appropriate to predict the scaling relationships observed (Hernández, 2000). Overall, maximum speeds and accelerations showed positive allometry whereas the timing and durations showed negative allometry. Thus, adults are faster than predicted resulting in relatively shorter durations and times to peak excursions. These results differ from those of Reilly (1995) concerning the larval stages of fire salamanders (*Salamandra salamandra*). In his study, Reilly (1995) examined the kinematics of eight salamander larvae on the day of birth and again 2 months later, just prior metamorphosis when their SVL had doubled. He found that the kinematics of feeding did not scale with size (Reilly, 1995). Our results also differ from those obtained by Deban and O'Reilly (2005) looking at the change in feeding kinematics in an ontogenetic series of hellbenders (*Cryptobranchus alleganiensis*). The authors compared the kinematics across ten individuals (three larvae and seven adults), ranging in size from 43 to 370 mm. They found that the kinematic variables mostly scaled according to the predictions of a Hill model (Deban and O'Reilly, 2005). Consequently, the impact of size on kinematics cannot be generalized across salamander species.

### Adults may generate a stronger suction flow and catch larger prey

The first phase of suction feeding involves buccal expansion which produces suction forces to bring prey into the mouth. This involves the rapid opening of the mouth, followed quasi-simultaneously by the depression of the hyobranchial apparatus, together creating an expansion of the oropharyngeal volume, and thus a drop in pressure that draws water and prey into the mouth (Lukanov et al., 2016; Heiss et al., 2013; Deban, 2003). The quantification of the hyoid depression is crucial as, most of the time, the mouth starts to close before maximum hyoid depression is reached. The depression of the hyobranchial apparatus ensures that water keeps flowing inward until buccal expansion reaches its maximum (Kucera et al., 2017; Deban, 2003). Our results show that peak gape (MG) and the peak depression of both anterior and posterior parts of the hyoid (Mhd1 and Mhd2) increase with size (Fig. 2A-C). Consequently, buccal expansion increases with size, conferring to adults the ability to capture larger prey (Fig. 2D). Indeed, strong correlations between the size of the prey and of the predator's mouth are often observed in other aquatic vertebrates like fish (Werner, 1974; Keast, 1985; Wainwright and Richard, 1995; Wainwright et al., 2007). In addition, larger prey are often more elusive (Wainwright et al., 2007) which requires a stronger suction flow. Here, we observe that maximum speeds and accelerations of mouth opening and hyobranchial depression increase with size (Fig. 3A,B,D,E,G,H). Interestingly, there is a clear shift between immature individuals and adults for the maximum accelerations of the opening of the mouth and of the depression of the anterior part of the hyobranchial apparatus (Fig. 3B and E). The slopes of these variables were respectively five and seven times higher in adults than in immature individuals (Fig. 3B and E). Conversely, the time to peak gape remains constant and is independent of size (slope=0.00±0.00; Fig. 3C) and the time to reach peak hyoid depression increases with size only at a slope of 0.01 cm s^−1^ (Fig. 3F and I). Thus, the speed of buccal expansion increases faster with size in adults compared to immature individuals.

**Fig. 1.**
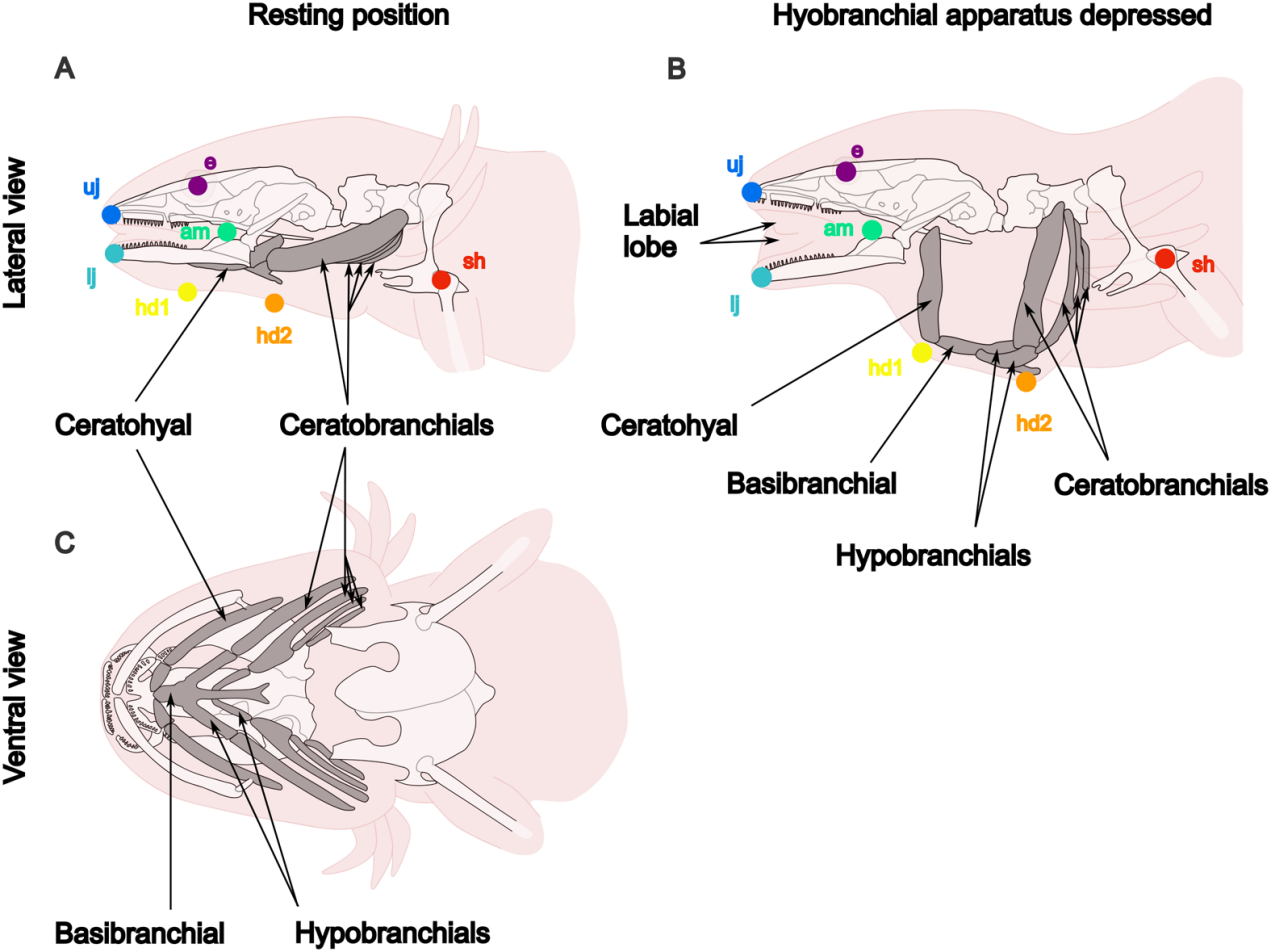
**Schematic diagram of the cranial structure of a larval *A. mexicanum*.** The head is represented in resting position on the left (A) and with the hyobranchial apparatus depressed on the right (B). In the lateral view, the landmarks correspond to the landmarks used to track the movements of cranial elements during suction feeding. The landmarks were the eye (e), the tip of the upper jaw (uj), the tip of the lower jaw(lj), the angle of the mouth (am), the point where the connection between the ceratohyals and the basibranchial protrudes ventrally (hd1), the point where the connection between the ceratobranchials and the basibranchial protrudes ventrally (hd2) and the shoulder (sh). A and B are modified from Deban (2003) and C is modified from Lauder and Shaffer (1985). In panel B, the labial lobes are visible. The labial lobes are flaps of skin located at the corner of the mouth between the upper and lower jaws. In *A. mexicanum* they are present in larvae, juveniles and adults.

**Fig. 2.**
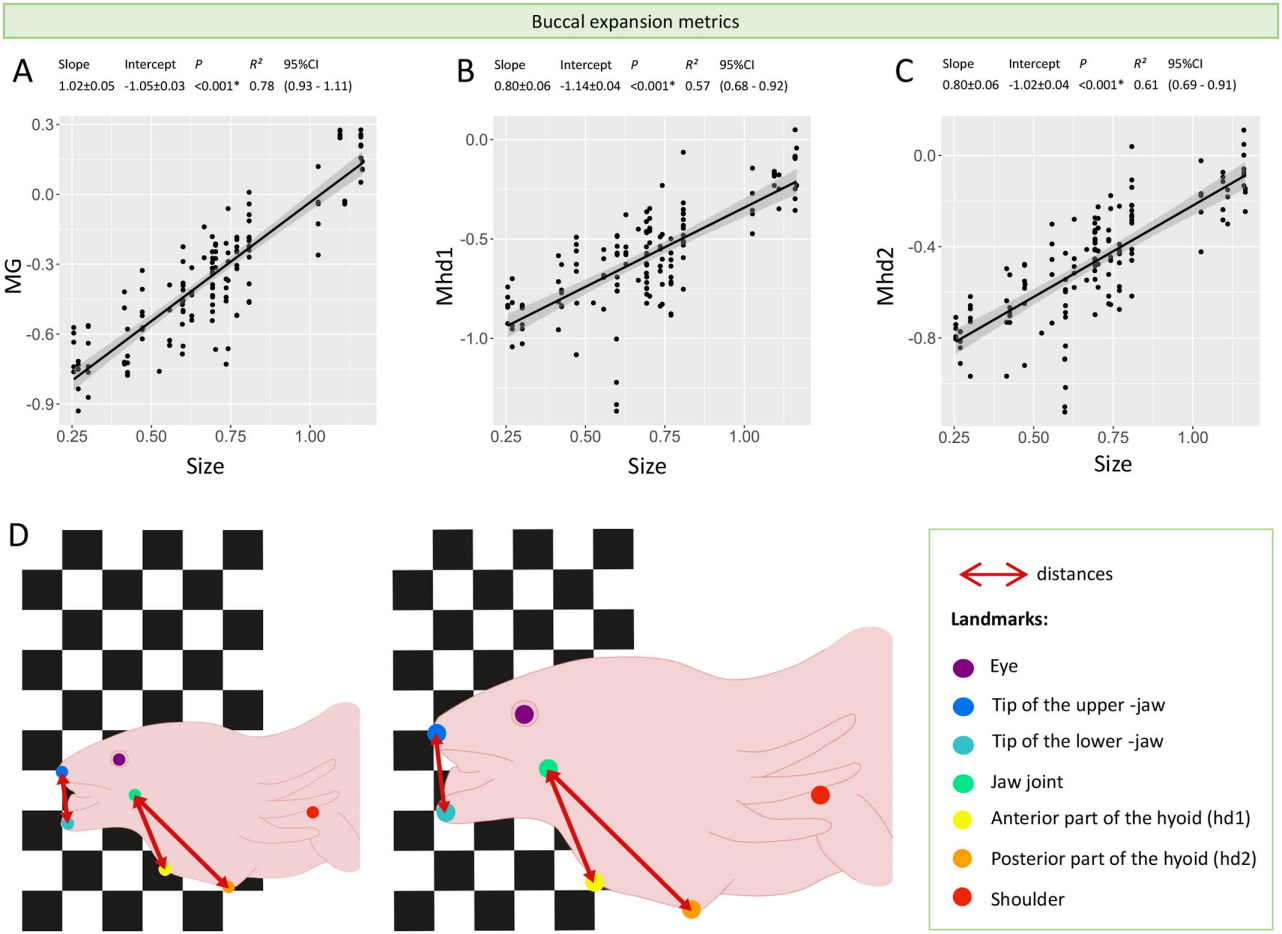
**Changes in buccal expansion metrics with size.** Maximum gape distance (MG), maximum depression distance of the anterior (Mhd1) and posterior (Mhd2) part of the hyoid are only impacted by size. Graphs A, B and C, and scheme D illustrate how these kinematic variables increase with size during buccal expansion. Size is approximated with snout–vent length (SVL). All values are in log10. Each point represents the kinematic value of the variable extracted in the corresponding video. * Denotes significance of the *P*-value meaning the linear relationship is significant. Maximum gape between upper and lower jaw, MG; maximum depression of the anterior part of the hyoid, Mhd1; maximum depression of the posterior part of the hyoid, Mhd2.

**Fig. 3.**
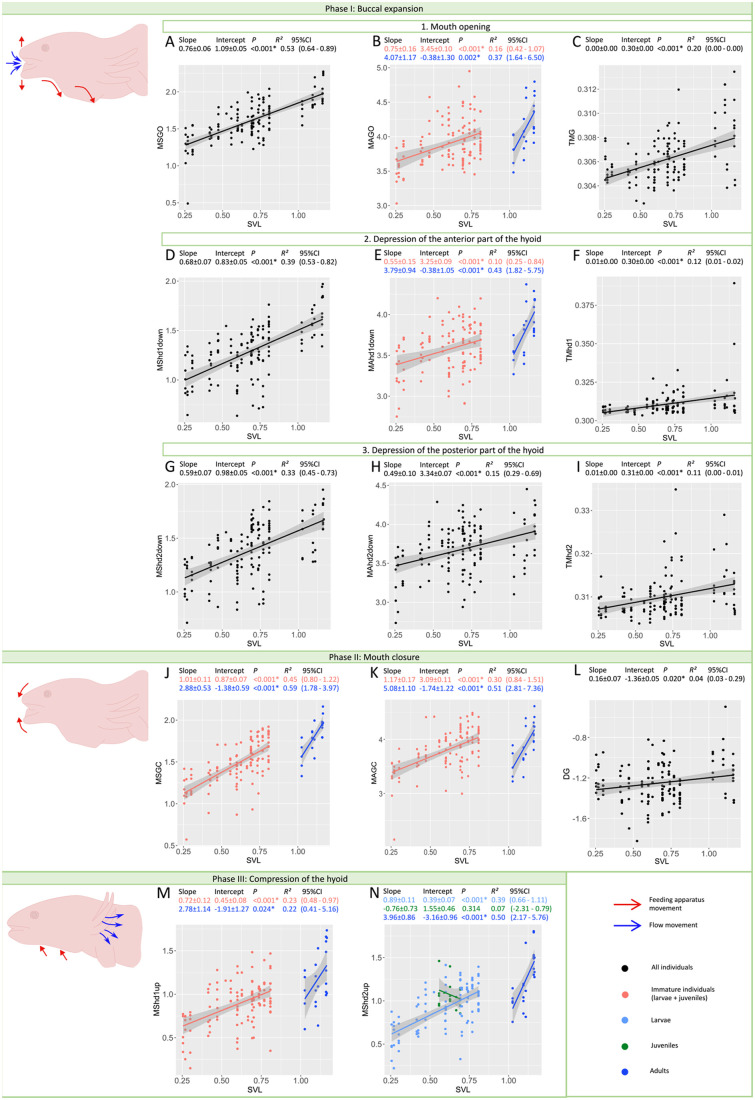
**Changes in maximum speeds and accelerations and associated timing with size and developmental stage through suction feeding.** Suction feeding is divided into three phases. Phase I permits the animal to engulf water and prey inside the buccal cavity. It involves the opening of the mouth and the depression of the hyoid. Phase II overlaps with phase I, it concerns the closing of the mouth to ensure the capture. Phase III is the compression of the hyoid which permit to expel the water through the gill slit. All maximum speeds and accelerations increase with size (except in juveniles MShd2up) but MAGO, MAhd1down, MSGC, MAGC, MShd1up and MShd2up increase faster with size in adults than in immature individuals. By contrast, timings of buccal expansion remain quasi unchanged whatever the size (TMG) or increase at a very slow rate (TMhd1, TMhd2 and DG). Size is approximated with SVL. All values are in log10. Each point represents the kinematic value of the variable extracted in the corresponding video. * denotes significance of the *P*-value meaning the linear relationship is significant. Whether stages are separated or not depends on the results of the ANCOVAs. For abbreviations: see Table 1.

**Fig. 4.**
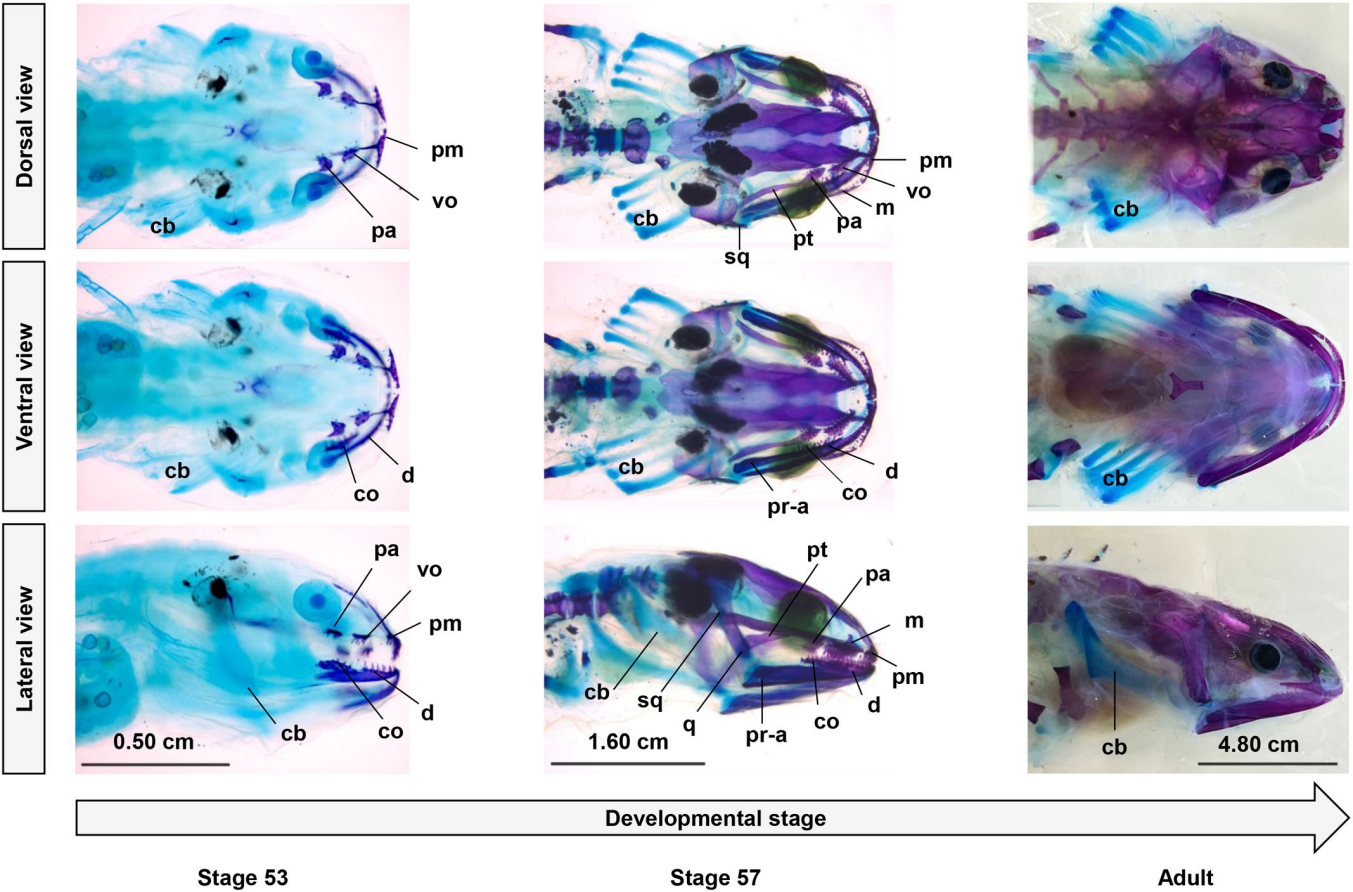
**Alcian Blue/Alizarin Red staining of specimens from stage 53 (snout–vent length, SVL: 2.2 cm), stage 57 (SVL: 2.2 cm), and an adult (SVL: 8 cm) of *A. mexicanum* in dorsal, ventral and lateral views, showing the degree of ossification of the feeding apparatus through ontogeny.** Calcified tissues appear red and cartilaginous tissues appear blue. The jaws are already partly ossified at stage 53, then ossification of the cranium is progressive and complete at adulthood. The hyobranchial apparatus and gills remain cartilaginous at all stages. cb, ceratobranchials elements of the hyobranchial apparatus, co, coronoid; d, dentary; m, maxilla; pa, palatine structure of the palatopterygoid; pm, premaxilla; pr-a, prearticular-angular (the two bones are fused); pt, pterygoid structure of the palotopterygoid; q, quadrate; sq, squamosal; vo, vomer.

To understand the consequences of such a shift, Wainwright and Day (2007) developed a mathematical model based on empirical data from feeding bluegill fish, *Lepomis macrochirus*. They found that three main forces are exerted on the prey during suction: drag force, acceleration reaction, and pressure gradient force (Wainwright and Day, 2007). With their simulations, they showed that the main force exerted on the prey during suction was the pressure gradient force, whether the prey was immobile, elusive, or attached to the substrate (Wainwright and Day, 2007). Their simulations also revealed that increasing the pressure gradient force was possible either temporally, by increasing the rate at which peak flow velocity is achieved by increasing the rate of expansion of the buccal cavity, or spatially, by decreasing the size of the mouth aperture (Wainwright and Day, 2007). The faster maximum speeds and accelerations in adult axolotls observed in our study may then correspond to a temporal way to increase the pressure gradient force allowing adults to generate a stronger suction flow. Moreover, in axolotls the retaining of a labial lobe (a flap of skin that joins the upper and lower jaws; see Fig. 1 and Movies 1-3) in adults may compensate for the increasing peak gape with size. Indeed, Van Wassenbergh and Heiss (2016) observed that the presence of labial lobes in the smooth newt (*Lissotriton vulgaris*) during the breeding season increased both suction distance and suction force by approximately 15%, notably by limiting the amount of flow from the lateral sides of the mouth.

### Adults may be more effective capturing prey

The second phase of feeding is mouth closure, ensuring the capture of the prey either inside the mouth or between the jaws of the predator. It starts at peak gape and ends when the jaws are closed. Our results revealed that the maximum speed of mouth closure increased with size but the slope was three times higher in adults than in immature individuals and the slope of the maximum acceleration during closing was four times higher in adults than in immature individuals (Fig. 3J and K). Yet, the slope of the duration of the gape cycle was only 0.16 (Fig. 3L) suggesting that the duration of mouth opening and closing remains rather invariant. Again, the shift in maximum speed and acceleration for mouth closing observed in adults is coherent with the hypothesis that adults may be more performant in capturing elusive prey (Holzman et al., 2008).

### Adults may generate a better unidirectional flow

The third phase involves buccal compression which expulses water from the oral cavity in a unidirectional manner (Lauder and Shaffer, 1985; Deban, 2003; Heiss and Grell, 2019). The compression is driven by the hyoid being pulled back to its resting position, accompanied by the opening of the gill slits (Deban, 2003). Our results show that, even when the hyobranchial apparatus returns to its resting position, the maximum speed of the anterior part of the hyoid increases with size but at a rate that is four times higher in adults than in immature individuals (Fig. 3M). Additionally, the maximum speed of the posterior part of the hyoid increases at a rate that is four times higher in adults than in larvae (Fig. 3N).

In adults, the maximum speeds reached by the hyobranchial apparatus increase more rapidly than in immature individuals. This could be because adults are expelling water more efficiently, which might compensate for the greater volume of water they engulf compared to immature individuals. A unidirectional flow of water through the mouth is considered advantageous when capturing elusive prey (Lauder and Shaffer, 1985). Indeed, individuals do not need to open the jaws to expel the excess water, reducing the possibility of prey to escape. It also creates a flow, drawing prey deeper into the throat. If the prey extends beyond the mouth, this flow can pull the prey as it is held between the jaws. In addition, the faster the predator expels the water, the faster it can depress the hyoid again to transport and swallow the prey. Adults, therefore, may be more efficient in swallowing prey.

### Do changes in anatomy at adulthood explain the observed shifts in performance?

We showed that size had an influence on all speed and acceleration variables of both the mouth and hyoid. However, the effect of size differed according to the developmental stage. Globally, the speed of movements increased three to seven times faster with size in adults. These data likely reflect the partial remodeling of the feeding apparatus that takes place during metamorphosis in axolotls. It has been shown that their cranial muscles are functional at stage 45, right after hatching (Ziermann and Diogo, 2013). As all larvae of our sample are posterior to stage 45, the muscles implicated in the movements of the mouth and the hyobranchial apparatus are already differentiated and fully functional. However, changes in muscle mass and musculoskeletal anatomy through ontogeny are expected due to their partial metamorphosis. Iordansky (2001) reported such changes occurring in the skull and hyobranchial musculature during partial metamorphosis in several paedomorphic salamander species, notably in the axolotl. Interestingly, in the axolotl, he found that most of the myological characters he investigated retained the larval conditions, except for the *levator bulbi*, which is present in adults only, and the *depressor mandibulae*, which is intermediate between the larval and adult condition (Iordansky, 2001). The *depressor mandibulae* is the muscle allowing the opening of the mouth, and its maturation may thus be implicated with the shift in maximum acceleration of mouth opening observed in adults. Moreover, the cleared and stained specimens included in this study reveal drastic changes in the degree of ossification of the head, from stage 57 (late larval stage) to adulthood (Fig. 4). In late larvae, the feeding structures of the cranium and of the lower jaw are partly ossified, while adults display fully ossified structures (Fig. 4). Iordansky (2001) further found that the hyobranchial apparatus and the vomer retain the larval condition in adult *A. mexicanum* compared to adult Ambystomatidae that naturally undergo complete metamorphosis. In contrast the palate corresponds to the middle stage of metamorphosis. Apart from these differences, the rest of the skull remains similar to that observed in the adult condition of metamorphic Ambystomatidae species, yet slightly less ossified. Thus, even if they are paedomorphic, axolotls undergo a partial metamorphosis with some degree of remodeling of the skull and some feeding muscles when becoming adult. We hypothesize these changes may explain the shift in speeds and accelerations observed in adult axolotls.

Given the differences observed in speeds and accelerations slopes one can wonder whether they are associated with an ontogenetic shift in diet with adults feeding on larger and/or more elusive prey like fish or elusive aquatic insects. Unfortunately, the diet of wild axolotl remains poorly known. Only one mention of a shift in diet in wild axolotl was found in the literature (www.amphibiaweb.org) stating that young axolotls may feed on algae, whereas adults tend to eat aquatic insects. A change in habitat use may also be associated with this change in diet. In bluegill sunfish it has been shown that the increase in the flow rate and closing speed of the mouth during growth contributes to the ontogenetic change in habitat use, from the heavily vegetated coastal areas to more pelagic habitats (Holzman et al., 2008). In our study, even if adults and immature axolotls look globally similar and share the same environment because of their paedomorphic life cycle, they may not compete for the same food as larvae and may use different micro habitats, yet this remains to be tested.

To conclude, our study demonstrates that both size and developmental stage have an influence on suction feeding kinematics in the axolotl. Indeed, despite the fact that adults look externally similar to immature individuals, they differ in their feeding kinematics. The speed of most movements increases faster with size in adults compared to immature individuals. These differences in performance through ontogeny might be explained by the remodeling of the musculoskeletal system during the partial metamorphosis and may allow changes in diet. It would be of interest to perform longitudinal studies of suction feeding coupled to *in vivo* imaging as this would allow to determine when the shift in performance coincides with changes in anatomy. Moreover, to better characterize the movements of the feeding apparatus during suction, it may be relevant to use biplanar X-ray video recordings (Brainerd et al., 2010; Gatesy et al., 2010; Knörlein et al., 2016).

## MATERIALS AND METHODS

Animal handling and experiments were approved by the Comité d'Ethique Cuvier project 2024-68-134 and follow the directive 2010/63/EU for animal research within the European Union.

Twenty-five specimens of the axolotl, *A. mexicanum* (Shaw and Nodder, 1798) belonging to different ontogenetic stages were used in this study. All the specimens came from breeding colonies in French laboratories (UMR 7221 and UMR 7179, CNRS-MNHN, Paris, France), and the Amphibian Foundation, in Atlanta, GA, USA. Developmental stages were assessed using detailed descriptions in the literature (Schreckenberg and Jacobson, 1975; Bordzilovskaya and Dettlaff, 1979; Nye et al., 2003). Here, we use three categories of developmental stages: larvae, juveniles, and adults. For the larval stages, only actively feeding individuals were used. The degree of limb development was used as a criterion to determine the developmental stage of the larvae (Table S1). Juveniles were differentiated from larval stages using the thickness of the hind limb as a benchmark. In juveniles, limb development is completed and individuals show stronger and longer hind limbs than forelimbs. Finally, the adult group was composed of sexually mature individuals. Following these definitions, data were collected for sixteen larvae, four juveniles, and five adults (Table S1). In some of the analysis, larvae and juveniles were combined into one group of ‘immature’ individuals.

### Experimental setup

Individuals were placed in a transparent plexiglass or glass experimental tank filled with water at room temperature (18°C). A 0.5×0.5 cm checkerboard was put in the tank to serve as a scale. Two infrared lights (dedolight LEDs) were used to record videos under low light conditions without disturbing the animals. A high-speed Phantom Miro R 311 camera (1000 fps) with a Nikon 50 mm lens was placed at the front of the tank and was connected to a computer with the PCC (Phantom Camera Control) software (version 3.6). All the videos were recorded in ‘cine’ format.

The food offered to the animals was adapted to their size and feeding habits in the respective laboratories. Smaller axolotl larvae around 2 cm were fed live daphnia. Adults were offered pieces of live earthworms (between 1 and 2.5 cm long). The food offered to other larvae and juveniles consisted of either pieces of earthworm (between 1 and 2.5 cm), live brown worms (≈1 cm long and 1 mm wide), or dead bloodworms (≈1 cm long and 1 mm wide). The difference between prey types should not dramatically impact the kinematics of suction feeding as long as prey size relative to head size remains constant (Reilly and Lauder, 1989). Moreover, all prey used in our study are static prey. Some animals were fed with tweezers as they ate more willingly if food was moving. Individuals were fed until satiated. Only feeding sequences in which the animals were positioned in lateral view throughout the feeding sequence were retained for this study.

### Extraction of size for each individual

For each individual, SVL was extracted from several frames from each video using ImageJ (Schneider et al., 2012). The SVL attributed to a given individual corresponded to the average of the SVL measurements of the different videos of that individual.

### Extraction of two-dimensional point coordinates over time

The beginning of a feeding bout was defined as the time when the mouth started to open, and the end as the time when the hyoid apparatus had returned to its initial position or stopped its movement. Seven landmarks were used to quantify the movements of the head during suction feeding (Fig. 1):

- the eye (e);
- the tip of the upper jaw (uj);
- the tip of the lower jaw (lj);
- the angle of the mouth (am);
- the anterior point of the hyoid apparatus (hd1), which corresponds to the connection between the ceratohyals and the basibranchial (Deban, 2003; Lauder and Shaffer, 1985);
- the posterior point of the hyoid apparatus (hd2), which corresponds to the connection between the ceratobranchials and the basibranchial (Deban, 2003; Lauder and Shaffer, 1985);
- the shoulder (sh).

Landmark tracking was performed using the deep-learning software DeepLabCut version 2.3.8 (Mathis et al., 2018; Nath et al., 2019). For each video, 50 frames were labelled manually to train a neural network through 500,000 iterations initially. DeepLabCut predictions were verified using the labelled videos. For videos where predictions were not accurate, new images were extracted and labelled and new iterations were run to improve the training. This process was repeated until predictions were accurate.

Once the two-dimensional coordinates of the landmarks over time were obtained, they were exported in ‘csv’ format. R version 4.4.2 [R Core Team, 2024 (https://www.R-project.org/)] was used to convert times (in s) and distances (in cm) and to calculate variables concerning the movement of the jaws and hyoid apparatus. Curves of x and y over time were smoothed with 80 degrees of freedom using the ‘smooth.spline’ function (R Core Team, 2024). From the smoothed (x, y) coordinates, the following kinematic variables were calculated:

- the distance between the tips of the upper and lower jaws;
- the speed and acceleration of mouth opening and closing movements;
- the angle between the upper jaw, the jaw joint, and the lower jaw;
- the distance between the hyoid points and the jaw joint;
- the speed and acceleration of hyoid depression;
- the angle of the head formed between the upper jaw, eye and shoulder points.

Distances were calculated using the formula: 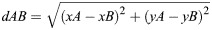, *dAB* being the distance between points A and B. Speeds were calculated by deriving distance versus time using ‘diff’ function (R Core Team, 2024). Accelerations were calculated by deriving speed versus time using ‘diff’ function (R Core Team, 2024). Angles were calculated by applying Al-Kashi's theorem, according to which: 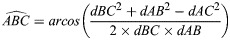, with *dBC*, *dAB*, and *dAC* being, respectively, the distance between points B and C, A and B, A and C. Radians were converted to degrees by multiplying by 180°/π.

### Kinematic variables

For each video, 25 kinematic variables were extracted and used in the subsequent analysis to describe the movements of the jaws and hyoid apparatus. The kinematic variables and their associated abbreviations are listed in Table 1.

### Statistical analyses

All the statistical analysis were performed using R version 4.4.2 (R Core Team, 2024). Prior to analyses, all variables were log_10_ transformed.

#### Size differences between developmental stages

To test whether larval, juvenile, and adult stages in our dataset differed in size, an analysis of variance was performed using the ‘aov’ function in the ‘stats’ package (R Core Team, 2024). Next, a multiple comparison with Bonferroni adjustment was performed using the ‘emmeans’ function from the ‘emmeans’ package (Lenth et al., 2024) to determine which groups differed from one another.

#### Scaling

For each kinematic variable, the peak performance per individual (i.e. largest gape, fastest jaw opening, etc.) was regressed against SVL using the ‘lm’ function (R Core Team, 2024). We tested for allometry by comparing the slopes predicted by the Hill model (1950) to the slopes obtained based on our dataset (Table 2). The relationships were considered to exhibit negative allometry if the predicted slope from the Hill model was above the upper 95% confidence interval of the regression slope. Conversely, the relationships were considered to exhibit positive allometry if the predicted slope was below the lower 95% confidence interval of the regression slope.

**Table 2.** Scaling of feeding kinematics

Type of variable	Kinematic variables	Slope	Intercept	*P*	*R^2^*	95%CI	Hill predictions	Type of allometry
Metric (cm)	MG	0.98±0.09	−0.92±0.07	<0.001*	0.83	(0.79-1.17)	1	Isometry
	Mhd1	0.86±0.12	−1.03±0.09	<0.001*	0.70	(0.61-1.10)	1	Isometry
	Mhd2	0.80±0.12	−0.90±0.09	<0.001*	0.67	(0.56-1.05)	1	Isometry
Angle (°)	MGA	0.15±0.11	1.63±0.08	0.166	0.08	(−0.07-0.37)	0	Isometry
	MHA	0.00±0.02	2.21±0.01	0.791	0.00	(−0.04-0.03)	0	Isometry
Speed (cm s^−1^)	MSGO	0.72±0.09	1.32±0.07	<0.001*	0.73	(0.53-0.90)	0	Positive
	MSGC	0.72±0.09	1.21±0.07	<0.001*	0.73	(0.53-0.91)	0	Positive
	MShd1down	0.67±0.12	1.03±0.08	<0.001*	0.59	(0.43-0.91)	0	Positive
	MShd2down	0.67±0.09	1.13±0.06	<0.001*	0.72	(0.49-0.85)	0	Positive
	MShd1up	0.80±0.14	0.63±0.10	<0.001*	0.59	(0.51-1.09)	0	Positive
	MShd2up	0.71±0.14	0.72±0.10	<0.001*	0.54	(0.42-0.99)	0	Positive
Acceleration (cm s^−2^)	MAGO	0.69±0.21	3.86±0.15	0.003*	0.33	(0.26-1.11)	−1	Positive
	MAGC	0.63±0.20	3.67±0.14	0.004*	0.31	(0.22-1.04)	−1	Positive
	MAhd1down	0.54±0.13	3.57±0.09	<0.001*	0.43	(0.27-0.80)	−1	Positive
	MAhd2down	0.65±0.11	3.56±0.08	<0.001*	0.59	(0.42-0.89)	−1	Positive
Timing (s)	TMG	0.00±0.00	0.30±0.00	0.020*	0.21	(0.00-0.01)	1	Negative
	Thd1	0.00±0.00	0.30±0.00	0.016*	0.23	(0.00-0.00)	1	Negative
	Thd2	0.00±0.00	0.30±0.00	0.588	0.01	(0.00-0.00)	1	Negative
	TMhd1	0.03±0.01	0.29±0.01	0.004*	0.31	(0.01-0.06)	1	Negative
	TMhd2	0.01±0.00	0.31±0.00	0.016*	0.23	(0.00-0.02)	1	Negative
	TMHA	−0.06±0.03	0.38±0.02	0.065*	0.14	(−0.12-0.00)	1	Negative
Duration (s)	DG	0.27±0.21	−1.27±0.15	0.214	0.07	(−0.17-0.71)	1	Negative
	Dhd1	0.15±0.20	−0.56±0.15	0.454	0.02	(−0.26-0.57)	1	Negative
	Dhd2	0.09±0.19	−0.53±0.13	0.631	0.01	(−0.29-0.48)	1	Negative
	PCD	0.07±0.20	−0.48±0.14	0.711	0.01	(−0.33-0.48)	1	Negative

*Denotes a significant linear relationship between the kinematic variable and size.

#### Differences between ontogenetic stages (Fig. S3)

##### Splitting the variables into two categories depending on the significance of the interaction term (Fig. S3A)

For each individual, several feeding sequences were analyzed. To take this into account, we used a linear mixed model using the ‘lmer’ function of the lmerTest package (Kuznetsova et al., 2017) where the individual was implemented as a random effect. These mixed models also took into account the interaction term between size and developmental stage and used a maximum likelihood method (ML). Type II ANCOVAs with Satterthwaite's method, were performed on these models, using the ‘anova’ function of the package ‘lmerTest’ (Kuznetsova et al., 2017). These type II ANCOVAs identify whether the kinematic variables are impacted by (1) size, (2) developmental stage, or (3) an interaction (joint effect) of both size and stage. Kinematic variables were then split into two groups, a first category with variables for which the interaction was not significant and the other category for which the interaction term was significant.

##### Variables with no significant interaction between size and developmental stage (Fig. S3B)

For the kinematic variables for which the interaction term was not significant (i.e. the metrics ‘MG’, ‘Mhd1’, ‘Mhd2’; the durations ‘DG’, ‘Dhd1’, ‘Dhd2’, ‘PCD’, the timings ‘TMG’, ‘TMHA’, ‘Thd1’, ‘TMhd1’, ‘TMhd2’; the angles ‘MGA’ and ‘MHA’; the speeds ‘MSGO’, ‘MShd1down’, ‘MShd2down’, and the acceleration ‘MAhd2down’; Table 3), the interaction term between size and development was removed from the mixed model to increase the statistical power of the analyses. The effect of size and developmental stage was then tested using type II ANCOVAs (‘anova’ function of package ‘lmerTest’; Kuznetsova et al., 2017). Next, differences between groups (larvae versus adults, juveniles versus adults, larvae versus juveniles) due to developmental stage were tested by performing post-hoc tests with Tukey adjustment on the new linear mixed model using the ‘emmeans’ function of the ‘emmeans’ package (Lenth et al., 2024). Last, the differences between groups due to size were tested by performing post-hoc tests with Tukey adjustment using the ‘emmeans’ function of the ‘emmeans’ package (Lenth et al., 2024) on a linear mixed model that only took into account the developmental stage.

**Table 3.** Results of the ANCOVAs when testing the significance of the interaction term between size (SVL) and developmental stage

	Kinematic variable	*F*	*P*
Mouth cycle	MG	2.119	0.144
	TMG	0.889	0.425
	MGA	1.056	0.365
	DG	1.898	0.179
	MSGO	2.208	0.114
	MAGO	3.926	0.022*
	MSGC	3.293	0.048*
	MAGC	5.419	0.012*
Head movements	MHA	0.449	0.643
	TMHA	0.163	0.850
Hyoid I cycle	Thd1	1.601	0.205
	Mhd1	0.146	0.865
	TMhd1	2.088	0.128
	Dhd1	0.506	0.610
	MShd1down	1.719	0.198
	MAhd1down	4.112	0.036*
	MShd1up	3.824	0.024*
Hyoid II cycle	Thd2	3.732	0.026*
	Mhd2	0.043	0.958
	TMhd2	2.146	0.121
	Dhd2	0.690	0.511
	MShd2down	0.610	0.549
	MAhd2down	3.000	0.053
	MShd2up	9.017	<0.001*
Cycle duration	PCD	0.761	0.480

*Denotes a significant difference.

**Table 4.** Results of the ANCOVAs for the category without significant interaction terms testing the effect of size and developmental stage

	Kinematic variable	Size		Developmental stage	
		*F*	*P*	*F*	*P*
Mouth cycle	MG	84.404	<0.001*	1.374	0.278
	TMG	0.707	0.411	0.764	0.478
	MGA	0.287	0.600	0.786	0.470
	DG	0.059	0.811	1.987	0.168
	MSGO	66.582	<0.001*	0.056	0.946
Head movements	MHA	2.415	0.137	3.388	0.052
	TMHA	5.659	0.030*	0.946	0.405
Hyoid I cycle	Thd1	0.740	0.391	2.973	0.054
	Mhd1	13.825	0.001*	1.622	0.219
	TMhd1	5.098	0.026*	0.212	0.809
	Dhd1	1.019	0.326	3.590	0.046*
	MShd1down	13.156	0.002*	1.141	0.339
Hyoid II cycle	Mhd2	29.297	<0.001*	0.931	0.409
	TMhd2	7.017	0.009*	0.265	0.768
	Dhd2	0.324	0.576	4.245	0.028*
	MShd2down	24.551	<0.001*	0.948	0.400
	MAhd2down	20.600	<0.001*	1.838	0.163
Cycle duration	PCD	0.775	0.390	3.202	0.062

*Denotes a significant difference.

**Table 5.** Results of the ANCOVAs for the different combinations of groups with significant interaction terms

	Kinematic variable	Group compared	Size (SVL)		Developmental stage		Size×developmental stage	
			*F*	*P*	*F*	*P*	*F*	*P*
Mouth cycle	MAGO	L-A	26.452	<0.001*	8.964	0.003*	8.251	0.005*
		J-A	6.413	0.048*	4.132	0.093	2.286	0.185
		L-J	20.891	<0.001*	0.003	0.957	0.001	0.977
	MSGC	L-A	73.700	<0.001*	5.977	0.021*	4.998	0.033*
		J-A	22.476	<0.001*	18.818	<0.001*	13.944	<0.001*
		L-J	66.487	<0.001*	0.635	0.432	0.764	0.389
	MAGC	L-A	28.834	<0.001*	7.918	0.012*	6.330	0.022*
		J-A	6.398	0.039*	21.199	0.003*	17.499	0.004*
		L-J	27.335	<0.001*	3.155	0.092	3.350	0.083
Hyoid I cycle	MAhd1down	L-A	10.322	0.008*	6.837	0.020*	6.763	0.020*
		J-A	3.106	0.127	6.900	0.039*	5.454	0.057
		L-J	8.446	0.017*	1.776	0.203	1.483	0.243
	MShd1up	L-A	35.383	<0.001*	4.762	0.031*	4.409	0.038*
		J-A	2.726	0.107	8.330	0.006*	7.503	0.009*
		L-J	35.014	<0.001*	3.219	0.075	2.992	0.086
Hyoid II cycle	Thd2	L-A	0.757	0.386	7.161	0.008*	7.527	0.007*
		J-A	2.582	0.149	4.560	0.068	3.235	0.112
		L-J	1.568	0.213	0.020	0.888	0.032	0.859
	MShd2up	L-A	70.555	<0.001*	14.973	<0.001*	13.754	<0.001*
		J-A	11.967	0.001*	23.066	<0.001*	17.710	<0.001*
		L-J	63.285	<0.001*	4.002	0.049*	3.243	0.074

L-A, comparison between larvae and adults; J-A, comparison between juveniles and adults; L-J, comparison between larvae and juveniles; I-A for comparison between immature individuals and adults.

*Denotes a significant difference.

##### Variables with significant interaction terms between size and developmental stage (Fig. S3C)

For kinematic variables for which the interaction term was significant (i.e. the times ‘Thd2’, the speeds ‘MSGC’, ‘MShd1up’, ‘MShd2up’, the accelerations ‘MAGO’, ‘MAGC’, ‘MAhd1down’; Table 3) the impact of size on kinematics differed between developmental stages. Thus, new mixed models taking into account the interaction term between size and developmental stages and using a maximum likelihood method were fitted for each pair of developmental stages (larvae versus adult, juveniles versus adult, larvae versus juveniles). Next, type II ANCOVAs were performed on these models to test whether the interaction term remained significant or whether only size or developmental stage were significant.

##### Linear regressions

Linear regressions (‘lm’ function of R; R Core Team, 2024) of kinematic variables on snout-vent length were performed to illustrate the main results of the type II ANCOVAs. Linear regressions were performed either across all individuals, either for immature and adult individuals, or for the three developmental stages separately (see Table S4). The regressions were plotted using ‘ggplot’ function of the ‘ggplot2’ package (Wickham, 2016).

All the R-script are available on Git Hub, see data and resource availability.

##### Assessment of the degree of ossification of the feeding apparatus through ontogeny

To assess the anatomical changes and mineralization degree of the hard tissues of the feeding apparatus throughout ontogeny, three specimens belonging to stage 53, stage 57 and adults were stained following the Alcian Blue, Alizarin Red method described in the literature (Riquelme-Guzmán and Sandoval-Guzmán, 2023). These specimens came from the breeding colony of the PHYMA laboratory (UMR 7221, CNRS-MNHN, Paris, France).

#### AI used for this manuscript

ChatGPT (OpenAI, 2021), a language model based on the generative pre-trained transformer (GPT) architecture was used for writing part of the R scripts.

## Supplementary Material

10.1242/biolopen.061860_sup1 Supplementary information

## References

[BIO061860C1] Ashley, M. A., Reilly, S. M. and Lauder, G. V. (1991). Ontogenetic scaling of hindlimb muscles across metamorphosis in the tiger salamander, *Ambystoma tigrinum*. *Copeia* 1991, 767-776. 10.2307/1446404

[BIO061860C2] Ashley-Ross, M. A. (1994). Metamorphic and speed effects on hindlimb kinematics during terrestrial locomotion in the salamander *Dicamptodon tenebrosus*. *J. Exp. Biol.* 193, 285-305. 10.1242/jeb.193.1.285 9317817

[BIO061860C3] Bordzilovskaya, N. P. and Dettlaff, T. A. (1979). Table of stages of the normal development of axolotl embryos and the prognostication, of timing of successive developmental stages at various temperatures. *Axolotl Newslett.* 7, 2-22.

[BIO061860C4] Brainerd, E. L., Baier, D. B., Gatesy, S. M., Hedrick, T. L., Metzger, K. A., Gilbert, S. L. and Crisco, J. J. (2010). X-ray reconstruction of moving morphology (XROMM): precision, accuracy and applications in comparative biomechanics research. *J. Exp. Zool.* 313A, 262-279. 10.1002/jez.589 20095029

[BIO061860C5] Brown, J. H., West, G. B. and Enquist, B. J. (2000). Scaling in biology: patterns and processes, causes and consequences. In *Scaling in Biology* (ed. J. H. Brown and G. B. West), pp. 1-24. Oxford, NY: Oxford University Press.

[BIO061860C6] Carrier, D. (1996). Ontogenetic limits on locomotor performance. *Physiol. Zool.* 69, 467-488. 10.1086/physzool.69.3.30164211

[BIO061860C7] China, V. and Holzman, R. (2014). Hydrodynamic starvation in first-feeding larval fishes. *Proc. Natl. Acad. Sci. USA* 111, 8083-8088. 10.1073/pnas.1323205111 24843180 PMC4050599

[BIO061860C8] Cook, A. (1996). Ontogeny of feeding morphology and kinematics in juvenile fishes: a case study of the cottid fish *clinocottus analis*. *J. Exp. Biol.* 199, 1961-1971. 10.1242/jeb.199.9.1961 9319872

[BIO061860C9] Deban, S. M. (2003). Constraint and convergence in the evolution of salamander feeding. In *Vertebrate Biomechanics and Evolution* (ed. V. L. Bels, J.-P. Gasc and A. Casinos), pp. 161-178. Bios Oxford.

[BIO061860C11] Deban, S. M. and O'Reilly, J. C. (2005). The ontogeny of feeding kinematics in a giant salamander *Cryptobranchus alleganiensis*: Does current function or phylogenetic relatedness predict the scaling patterns of movement? *Zoology* 108, 155-167. 10.1016/j.zool.2005.03.006 16351963

[BIO061860C12] Ericsson, R., Cerny, R., Falck, P. and Olsson, L. (2004). Role of cranial neural crest cells in visceral arch muscle positioning and morphogenesis in the Mexican axolotl, *Ambystoma mexicanum*. *Dev. Dyn.* 231, 237-247. 10.1002/dvdy.20127 15366001

[BIO061860C13] Ferry-Graham, L. A. (1998). Effects of prey size and mobility on prey-capture kinematics in leopard sharks *triakis semifasciata*. *J. Exp. Biol.* 201, 2433-2444. 10.1242/jeb.201.16.2433 9679105

[BIO061860C14] Gatesy, S. M., Baier, D. B., Jenkins, F. A. and Dial, K. P. (2010). Scientific rotoscoping: a morphology-based method of 3-D motion analysis and visualization. *J. Exp. Zool.* 313A, 244-261. 10.1002/jez.588 20084664

[BIO061860C15] Heiss, E. and Grell, J. (2019). Same but different: aquatic prey capture in paedomorphic and metamorphic Alpine newts. *Zool. Lett.* 5, 1-12. 10.1186/s40851-019-0140-4 PMC6660708 31372238

[BIO061860C16] Heiss, E., Natchev, N., Gumpenberger, M., Weissenbacher, A. and Van Wassenbergh, S. (2013). Biomechanics and hydrodynamics of prey capture in the Chinese giant salamander reveal a high-performance jaw-powered suction feeding mechanism. *J. R. Soc. Interface* 10, 20121028. 10.1098/rsif.2012.1028 23466557 PMC3627076

[BIO061860C17] Hernández, L. P. (2000). Intraspecific scaling of feeding mechanics in an ontogenetic series of zebrafish, *Danio rerio*. *J. Exp. Biol.* 203, 3033-3043. 10.1242/jeb.203.19.3033 10976040

[BIO061860C18] Hernandez, L. P. and Motta, P. J. (1997). Trophic consequences of differential performance: ontogeny of oral jaw-crushing performance in the sheepshead, Archosargus probatocephalus (*Teleostei, Sparidae*). *J. Zool.* 243, 737-756. 10.1111/j.1469-7998.1997.tb01973.x

[BIO061860C19] Herrel, A. and Gibb, A. C. (2006). Ontogeny of performance in vertebrates. *Physiol Biochem. Zool.* 79, 1-6. 10.1086/498196 16380923

[BIO061860C20] Hill, A. V. (1950). The dimensions of animals and their muscular dynamics. *Sci. Prog.* 38, 209-230.

[BIO061860C21] Hoff, K. S., Lannoo, M. J. and Wassersug, R. J. (1985). Kinematics of midwater prey capture by Ambystoma (Caudata: Ambystomatidae) larvae. *Copeia* 1985, 247-251. 10.2307/1444824

[BIO061860C22] Holzman, R., Collar, D. C., Day, S. W., Bishop, K. L. and Wainwright, P. C. (2008). Scaling of suction-induced flows in bluegill: morphological and kinematic predictors for the ontogeny of feeding performance. *J. Exp. Biol.* 211, 2658-2668. 10.1242/jeb.018853 18689419

[BIO061860C24] Iordansky, N. N. (2001). Jaw apparatus of the permanent-aquatic urodela: paedomorphosis, neoteny, and feeding adaptations. *Rus. J. Herpetol.* 8, 179-194.

[BIO061860C25] Keast, A. (1985). The piscivore feeding guild of fishes in small freshwater ecosystems. *Env. Biol. Fish.* 12, 119-129. 10.1007/BF00002764

[BIO061860C26] Knörlein, B. J., Baier, D. B., Gatesy, S. M., Laurence-Chasen, J. D. and Brainerd, E. L. (2016). Validation of XMALab software for marker-based XROMM. *J. Exp. Biol.* 219, 3701-3711. 27655556 10.1242/jeb.145383

[BIO061860C27] Kucera, F., Beisser, C. J. and Lemell, P. (2017). Size does matter – Intraspecific variation of feeding mechanics in the crested newt *Triturus dobrogicus* (Kiritzescu, 1903). *Acta Sci. Nat.* 5, 75-85. 10.2478/asn-2018-0011

[BIO061860C28] Kuznetsova, A., Brockhoff, P. B. and Christensen, R. H. B. (2017). lmerTest package: Tests in linear mixed effects models. *J. Stat. Softw.* 89, 1-26. 10.18637/jss.v082.i13

[BIO061860C30] Lauder, G. V. and Shaffer, H. B. (1985). functional morphology of the feeding mechanism in aquatic Ambystomatid salamanders. *J. Morphol.* 185, 297-326. 10.1002/jmor.1051850304 4057265

[BIO061860C31] Lenth, R. V., Bolker, B., Buerkner, P., Giné-Vázquez, I., Herve, M., Jung, M., Love, J., Miguez, F., Piaskowski, J., Rielb, H. et al. (2024). emmeans: estimated Marginal Means, aka Least-Squares Means. R package version 1.10.6, https://CRAN.R-project.org/package=emmeans.

[BIO061860C32] Lukanov, S., Tzankov, N., Handschuh, S., Heiss, E., Naumov, B. and Natchev, N. (2016). On the amphibious food uptake and prey manipulation behavior in the Balkan-Anatolian crested newt (*Triturus ivanbureschi*, Arntzen and Wielstra, 2013). *Zoology* 119, 224-231. 10.1016/j.zool.2016.02.002 27013264

[BIO061860C33] Mathis, A., Mamidanna, P., Cury, K. M., Abe, T., Murthy, V. N., Mathis, M. W. and Bethge, M. (2018). DeepLabCut: markerless pose estimation of user-defined body parts with deep learning. *Nat. Neurosci.* 21, 1281-1289. 10.1038/s41593-018-0209-y 30127430

[BIO061860C34] Nath, T., Mathis, A., Chen, A. C., Patel, A., Bethge, M. and Mathis, M. W. (2019). Using DeepLabCut for 3D markerless pose estimation across species and behaviors. *Nat. Protoc.* 14, 2152-2176. 10.1038/s41596-019-0176-0 31227823

[BIO061860C35] Nye, H. L. D., Cameron, J. A., Chernoff, E. A. G. and Stocum, D. L. (2003). Extending the table of stages of normal development of the axolotl: limb development. *Dev. Dyn.* 226, 555-560. 10.1002/dvdy.10237 12619140

[BIO061860C36] O'Reilly, J. C., Lindstedt, S. L. and Nishikawa, K. C. (1993). The scaling of feeding kinematics in toads (Anura: Bufonidae). *Am. Zool.* 33, 147A.

[BIO061860C37] Otten, E. (1983). The jaw mechanism during growth of a generalized Haplochromis species: *H. elegans* Trewavas 1933 (Pisces, Cichlidae). *Neth. J. Zool.* 33, 55-98. 10.1163/002829683X00048

[BIO061860C39] R Core Team. (2024). *R: A Language and Environment for Statistical Computing*. Vienna, Austria: R Foundation for Statistical Computing. https://www.R-project.org/.

[BIO061860C40] Reilly, S. M. (1995). The ontogeny of aquatic feeding behavior in *Salamandra salamandra*: stereotypy and isometry in feeding kinematics. *J. Exp. Biol.* 198, 701-708. 10.1242/jeb.198.3.701 9318449

[BIO061860C41] Reilly, S. M. and Lauder, G. V. (1989). Physiological bases of feeding behaviour in slamanders: do motor patterns vary with prey type? *J. Exp. Biol.* 141, 343-358. 10.1242/jeb.141.1.343

[BIO061860C42] Richard, B. A. and Wainwright, P. C. (1995). Scaling the feeding mechanism of largemouth bass (*micropterus salmoides*): kinematics of prey capture. *J. Exp. Biol.* 198, 419-433. 10.1242/jeb.198.2.419 9318056

[BIO061860C43] Riquelme-Guzmán, C. and Sandoval-Guzmán, T. (2023). Methods for studying appendicular skeletal biology in axolotls. In *Salamanders Methods and Protocols* (ed. A. W. Seifert and J. D. Currie), pp. 155-163. Humana Press. 10.1007/978-1-0716-2659-7_9 36272073

[BIO061860C44] Robinson, M. P. and Motta, P. J. (2002). Patterns of growth and the effects of scale on the feeding kinematics of the nurse shark (*Ginglymostoma cirratum*). *J. Zool.* 256, 449-462. 10.1017/S0952836902000493

[BIO061860C45] Rose, C. S. and Reiss, J. O. (1993). Metamorphosis and the vertebrate skull: ontogenetic patterns and developmental mechanism. In *The skull*, Vol. 1 (ed. J. Hanken and B. K. Hall), University of Chicago Press, pp. 289-346.

[BIO061860C46] Schmidt-Nielsen, K. (1984). *Scaling: Why is Animal Size So Important?* Cambridge, NY: Cambridge University Press.

[BIO061860C47] Schneider, C. A., Rasband, W. S. and Eliceiri, K. W. (2012). NIH Image to ImageJ: 25 years of image analysis. *Nat. Methods* 9, 671-675. 10.1038/nmeth.2089 22930834 PMC5554542

[BIO061860C48] Schreckenberg, G. M. and Jacobson, A. G. (1975). Normal Stages of Development of the Axolotl, *Ambystoma mexicanum*. *Dev. Biol.* 42, 391-400. 10.1016/0012-1606(75)90343-7 1167837

[BIO061860C49] Smirnov, S. V. and Vassilieva, A. B. (2004). Characteristics of Craniogenesis in the Axolotl (*Ambystoma mexicanum*: Ambystomatidae) and the role of thyroid hormones in its regulation. *Dokl. Biol. Sci.* 395, 121-123. 10.1023/B:DOBS.0000025235.17412.9a 15255140

[BIO061860C51] Van Wassenbergh, S. and Heiss, E. (2016). Phenotypic flexibility of gape anatomy fine-tunes the aquatic prey-capture system of newts. *Sci. Rep.* 7, 6-29277. 10.1038/srep29277 PMC4935879 27383663

[BIO061860C52] Werner, E. E. (1974). The fish size, prey size, handling time relation in several sunfishes and some implications. *J. Fish. Res. Bd Can.* 31, 1531-1536. 10.1139/f74-186

[BIO061860C53] Wickham, H. (2016). *ggplot2 – Elegant Graphics for Data Analysis*. NY: Springer-Verlag.

[BIO061860C54] Wainwright, P. C. and Day, S. W. (2007). The forces exerted by aquatic suction feeders on their prey. *J. R. Soc. Interface* 4, 553-560. 10.1098/rsif.2006.0197 17251163 PMC2373408

[BIO061860C58] Wainwright, P. C. and Richard, B. A. (1995). Predicting patterns of prey use from morphology with fishes. *Environ Biol Fishes*. 44, 97-113.

[BIO061860C55] Wainwright, P. C. and Shaw, S. S. (1999). Morphological basis of kinematic diversity in feeding sunfishes. *J. Exp. Biol.* 202, 3101-3110. 10.1242/jeb.202.22.3101 10539958

[BIO061860C56] Wainwright, P. C., Carroll, A. M., Colar, D. C., Day, S. W. and Higham, T. E. (2007). Suction feeding mechanics, performance, and diversity in fishes. *Integr. Comp. Biol.* 47, 96-106. 10.1093/icb/icm032 21672823

[BIO061860C57] Ziermann, J. and Diogo, R. (2013). Cranial muscle development in the model organism *Ambystoma mexicanum:* implications for tetrapod and vertebrate comparative and evolutionary morphology and notes on ontogeny and phylogeny. *Anat. Rec.* 296, 1031-1048. 10.1002/ar.22713 23650269

