# Supplementary material for: The impact of size and ontogeny on suction feeding kinematics in the axolotl (Ambystoma mexicanum)
Source: Biol Open. 2025 Jul 18;14(7):bio061860. doi: 10.1242/bio.061860 (PMC12309904; doi:10.1242/bio.061860)
Supplement: Supplementary information [file biolopen-14-061860-s1.pdf]

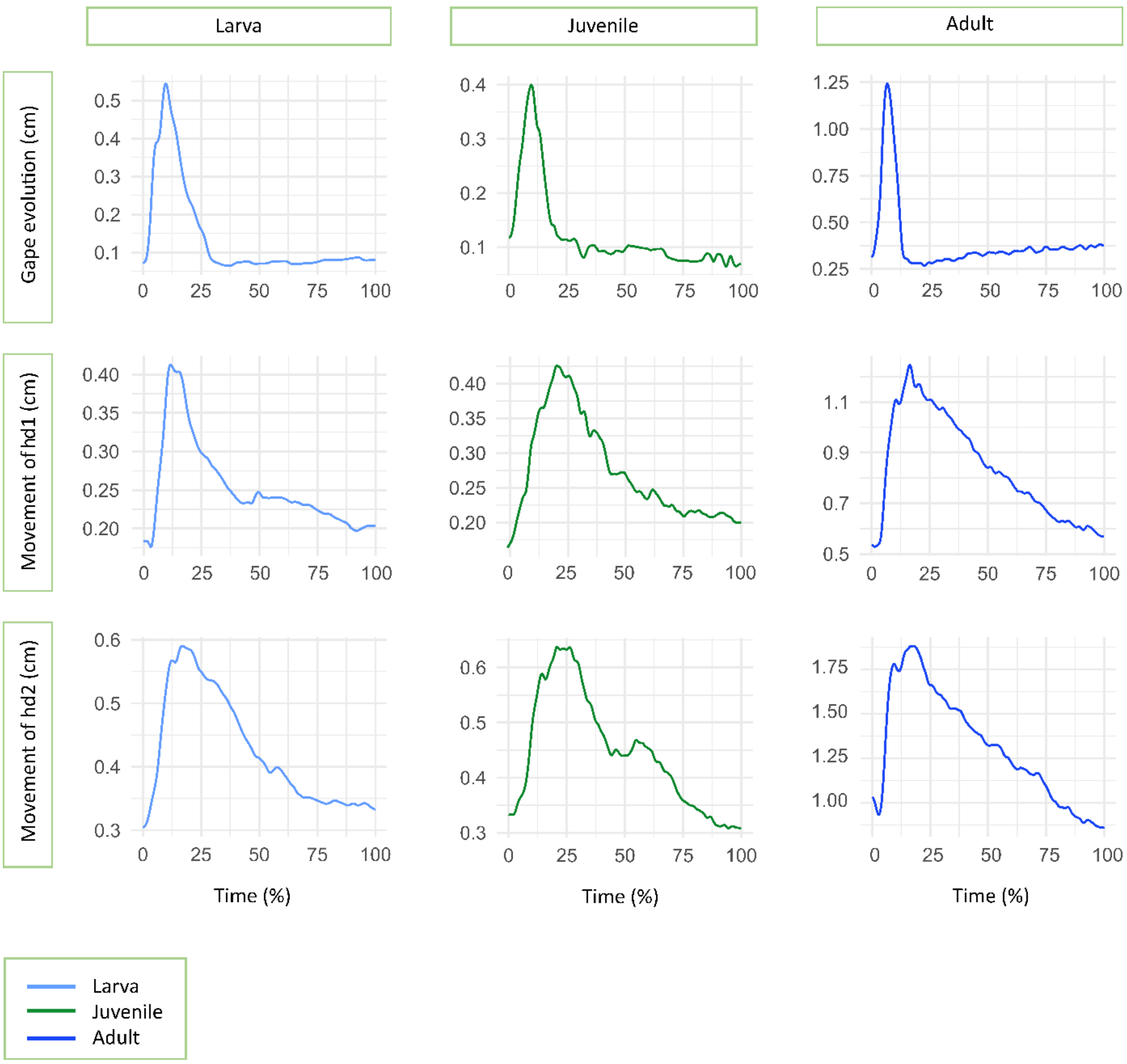


**Fig. S1. Changes in gape and the movement of the anterior the hyoid and posterior part of relative to overall cycle time (in %).**

Larval curves are extracted from sequence five of larva 2 (developmental stage= 54; SVL=2.96 cm); Juvenile curves are extracted from sequence 4 of juvenile 1 (SVL=4.24 cm); Adult curves are extracted from sequence 1 of adult 4 (SVL=10.61). The curves were smoothed using function "smooth.spline" of R with 80 degrees of freedom. Corresponding videos are M. S1, M. S2 and M. S3.

The time is express in percentage. 0% time correspond to the beginning of the suction feeding sequence, and 100% time correspond to the moment when the hyoid apparatus is back to its resting position.

Abbreviations: hd1, anterior part of the hyoid; hd2, posterior part of the hyoid;

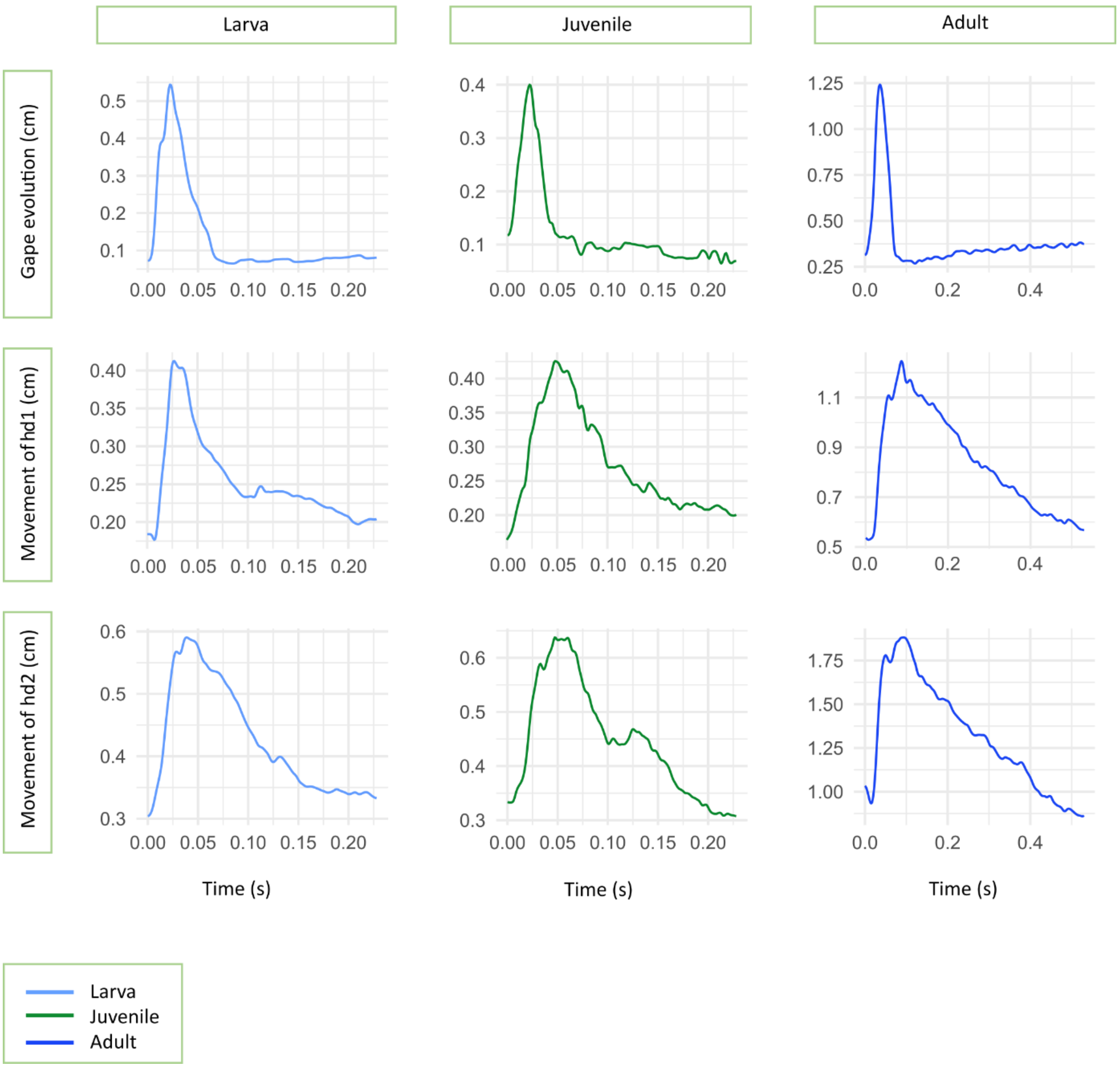


**Fig. S2. Changes in gape and the movement of the anterior and posterior part of the hyoid through time.**

Larval curves are extracted from sequence five of larva 2 (developmental stage= 54; SVL=2.96 cm); Juvenile curves are extracted from sequence 4 of juvenile 1 (SVL=4.24 cm); Adult curves are extracted from sequence 1 of adult 4 (SVL=10.61). The curves were smoothed using function “smooth.spline” of R with 80 degrees of freedom. Corresponding videos are M. S1, M. S2 and M. S3. The time axis starts when the mouth begins to open which correspond to the beginning of the suction feeding sequence, and ends when the hyoid apparatus is back to its resting position.

Abbreviations: hd1, anterior part of the hyoid; hd2, posterior part of the hyoid.

Biology Open • Supplementary information

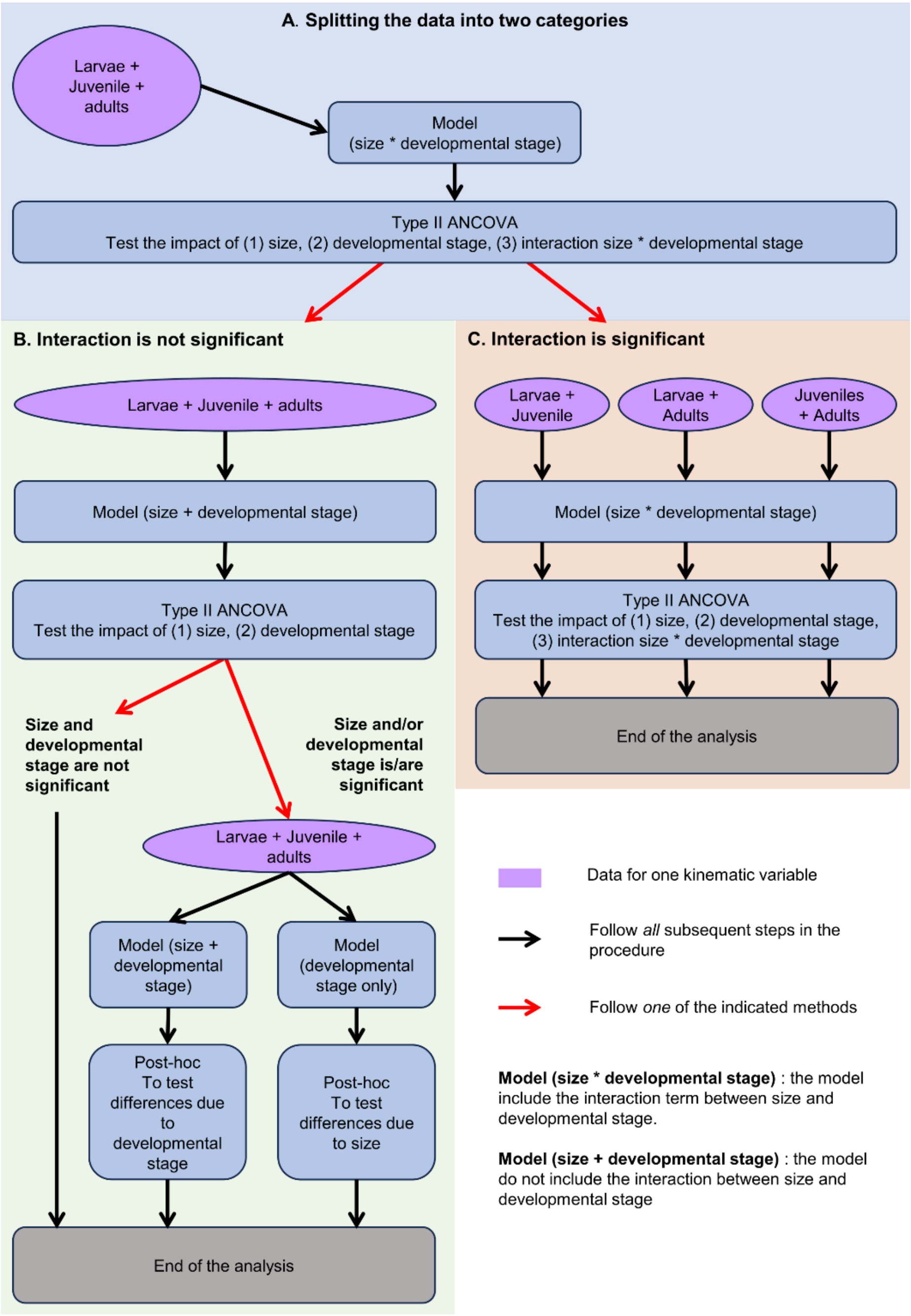


**Fig. S3. Overview of the statistical approach used to separate the effects of size, developmental stage, and their interaction throughout ontogeny.**

Panel A shows how kinematic variables are splitted into two categories based on whether the initial Type II ANCOVA detects a significant interaction between size and developmental stage.

Panel B outlines the steps followed when no significant interaction is detected.

Panel C outlines the steps followed when a significant interaction is detected.

All models are linear mixed models that account for repeated measurements (i.e., multiple feeding sequences per individual).

Biology Open • Supplementary information

**Table S1. Origin, size, and number of sequences for each individual analyzed.**

| Individuals | Provenance | SVL (cm) | Number of sequences |
|---|---|---|---|
| Larva 1 stage 53 | USA Amphibian foundation | 2.6 | 4 |
| Larva 2 stage 54 | USA Amphibian foundation | 2.96 | 6 |
| Larva 3 stage 55 | USA Amphibian foundation | 2 | 6 |
| Larva 4 stage 55 | USA Amphibian foundation | 1.86 | 5 |
| Larva 5 stage 55 | USA Amphibian foundation | 1.80 | 5 |
| Larva 6* stage 56 | Paris CNRS MNHN | 3.96 | 4 |
| Larva 7 stage 56 | Paris CNRS MNHN | 3.35 | 1 |
| Larva 8 stage 57 | Paris CNRS MNHN | 2.97 | 1 |
| Larva 9 stage 57 | USA Amphibian foundation | 2.66 | 5 |
| Larva 10 stage 57 | USA Amphibian foundation | 3.98 | 9 |
| Larva 11 stage 57 | USA Amphibian foundation | 5.04 | 6 |
| Larva 12* stage 57 | FRANCE CNRS MNHN | 6.43 | 14 |
| Larva 13 stage 57 | FRANCE CNRS MNHN | 4.93 | 11 |
| Larva 14 stage 57 | FRANCE CNRS MNHN | 5.88 | 9 |
| Larva 15 stage 57 | FRANCE CNRS MNHN | 5.44 | 6 |
| Larva 16 stage 57 | FRANCE CNRS MNHN | 5.52 | 5 |
| Juvenile 1 | USA Amphibian foundation | 4.24 | 5 |
| Juvenile 2 | USA Amphibian foundation | 3.61 | 5 |
| Juvenile 3 | USA Amphibian foundation | 4.92 | 5 |
| Juvenile 4 | USA Amphibian foundation | 4.64 | 2 |
| Adult 1 | FRANCE CNRS MNHN | 14.46 | 7 |
| Adult 2 | FRANCE CNRS MNHN | 12.64 | 3 |
| Adult 3 | FRANCE CNRS MNHN | 14.62 | 3 |
| Adult 4 | USA Amphibian foundation | 10.61 | 5 |
| Adult 5 | USA Amphibian foundation | 12.44 | 5 |

**Larva 6 and 12 are the same larva filmed at two different larval stages. It was maintained separate from other larvae because it was cannibalistic.*

**Table S2. Minimum, maximum, and average size of the different stages in our developmental data set.**

| Stage | Minimal SVL (cm) | Maximal SVL (cm) | Average SVL (cm) |
|---|---|---|---|
| Larvae | 1.80 | 6.43 | 3.84 |
| Juveniles | 3.61 | 4.92 | 4.35 |
| Adults | 10.61 | 14.62 | 13.00 |

Biology Open • Supplementary information

**Table S3. Results of the post-hoc tests with or without a correction for size.**

P-values are Tukey corrected.

| | Kinematic variable | Group compared | With correction for size | | | Without correction for size | | |
|---|---|---|---|---|---|---|---|---|
| | | | *df* | *t* | *P* | *df* | *t* | *P* |
| Mouth cycle | MG | Larvae – Adults | 28.2 | 1.456 | 0.327 | 28.1 | 6.938 | <0.001* |
| | | Juveniles – Adults | 32.1 | 1.084 | 0.531 | 28.6 | 4.268 | <0.001* |
| | | Larvae - Juveniles | 33.7 | 0.461 | 0.900 | 28.6 | 1.210 | 0.457 |
| | MSGO | Larvae – Adults | 14.2 | -0.320 | 0.945 | 28.8 | 4.824 | <0.001* |
| | | Juveniles – Adults | 28.6 | -0.225 | 0.973 | 29.8 | 3.126 | 0.011* |
| | | Larvae - Juveniles | 23.9 | -0.092 | 0.995 | 29.6 | 0.631 | 0.804 |
| Head movements | TMHA | Larvae – Adults | 26.9 | 0.962 | 0.607 | 29.4 | -1.133 | 0.502 |
| | | Juveniles – Adults | 31.3 | 1.237 | 0.441 | 31.4 | -0.210 | 0.976 |
| | | Larvae - Juveniles | 33.1 | -0.536 | 0.854 | 30.7 | -0.768 | 0.725 |
| Hyoid I cycle | Mhd1 | Larvae – Adults | 27.3 | 1.398 | 0.356 | 28.0 | 5.996 | <0.001* |
| | | Juveniles – Adults | 29.3 | 0.615 | 0.813 | 28.6 | 3.281 | 0.007* |
| | | Larvae - Juveniles | 30.4 | 1.186 | 0.470 | 28.6 | 1.526 | 0.294 |
| | TMhd1 | Larvae – Adults | 18.3 | 0.602 | 0.821 | 31.0 | 3.218 | 0.008* |
| | | Juveniles – Adults | 27.5 | 0.464 | 0.889 | 36.4 | 2.139 | 0.096 |
| | | Larvae - Juveniles | 31.6 | 0.118 | 0.992 | 34.4 | 0.282 | 0.957 |
| | Dhd1 | Larvae – Adults | 28.0 | 1.873 | 0.165 | 29.8 | 1.978 | 0.135 |
| | | Juveniles – Adults | 30.8 | 2.412 | 0.056 | 31.2 | 2.455 | **0.051** |
| | | Larvae - Juveniles | 32.0 | -0.997 | 0.584 | 30.9 | -1.155 | 0.489 |
| | MShd1down | Larvae – Adults | 26.8 | 1.240 | 0.441 | 28.7 | 5.324 | <0.001* |
| | | Juveniles – Adults | 31.2 | 0.671 | 0.782 | 30.3 | 3.128 | 0.011* |
| | | Larvae - Juveniles | 33.0 | 0.782 | 0.717 | 29.8 | 1.059 | 0.546 |
| Hyoid II cycle | Mhd2 | Larvae – Adults | 27.1 | 0.225 | 0.973 | 27.9 | 5.146 | <0.001* |
| | | Juveniles – Adults | 29.6 | -0.555 | 0.845 | 28.4 | 2.633 | 0.035* |
| | | Larvae - Juveniles | 30.8 | 1.239 | 0.440 | 28.5 | 1.531 | 0.292 |
| | TMhd2 | Larvae – Adults | 17.2 | -0.042 | 0.999 | 32.7 | 2.632 | 0.034* |
| | | Juveniles – Adults | 26.6 | 0.403 | 0.915 | 37.2 | 2.290 | 0.070 |
| | | Larvae - Juveniles | 30.8 | -0.693 | 0.769 | 35.6 | -0.414 | 0.910 |
| | Dhd2 | Larvae – Adults | 27.6 | 1.506 | 0.304 | 29.5 | 1.924 | 0.150 |
| | | Juveniles – Adults | 30.2 | 2.509 | 0.045* | 30.9 | 3.004 | 0.014* |
| | | Larvae - Juveniles | 31.5 | -1.715 | 0.216 | 30.6 | -1.860 | 0.168 |
| | MShd2down | Larvae – Adults | 23.5 | -0.634 | 0.803 | 28.1 | 3.630 | 0.003* |
| | | Juveniles – Adults | 29.0 | -1.180 | 0.474 | 29.7 | 1.713 | 0.217 |
| | | Larvae - Juveniles | 31.2 | 0.942 | 0.618 | 29.2 | 1.225 | 0.448 |
| | MAhd2down | Larvae – Adults | 17.4 | -1.635 | 0.258 | 30.2 | 1.976 | 0.136 |
| | | Juveniles – Adults | 26.8 | -1.755 | 0.204 | 32.5 | 0.802 | 0.704 |
| | | Larvae - Juveniles | 31.0 | 0.464 | 0.889 | 31.7 | 0.814 | 0.697 |

** Denotes a significant difference*
*Same abbreviations as for table 1.*

Biology Open • Supplementary information

**Table S4. Table of equations of the relationship of kinematic variables as a function of size (SVL) for the main results of the type II ANOVAs.** In the case where the variables were only impacted by size (metrics MG, Mhd1, Mhd2, timings TMhd1 and TMhd2, speeds MSGO, MShd1down, MShd2down, and acceleration MAhd2down) or in the case neither size or stage impacted the data (TMG and DG), overall linear regressions were performed across all individuals. In the case where the interaction term or stage was significant both between adults and larvae and between adults and juveniles but not between larvae and juveniles, larvae and juveniles were gathered as one group of 'immature' individuals. Linear regressions were performed for the two groups: immature and adult (MSGC, MAGC, Mahd1down, MShd1up). For MAGO the linear regression was also performed for he immature and adult group because larvae and juveniles were similar. For MShd2up, as the interaction terms between larvae and adults and larvae and juvenile were significant and as stage had a significant impact between larvae and juveniles, linear regressions were performed for each of the three groups separately (i.e. separately for larvae, juveniles, and adults).

| | Kinematic variable | Developmental stage | Slope | Intercept | *P* | $R^2$ | 95%CI |
|---|---|---|---|---|---|---|---|
| ➢ Variables that were not impacted by size or developmental stage | | | | | | | |
| Timing | TMG | All stages | 0.00 ± 0.00 | 0.30 ± 0.00 | <0.001* | 0.20 | (0.00 - 0.00) |
| Duration | DG | All stages | 0.16 ± 0.07 | -1.36 ± 0.05 | 0.020* | 0.04 | (0.03 - 0.29) |
| ➢ Variables that were only impacted by size | | | | | | | |
| Metrics | MG | All stages | 1.02 ± 0.05 | -1.05 ± 0.03 | <0.001* | 0.78 | (0.93 - 1.11) |
| | Mhd1 | All stages | 0.80 ± 0.06 | -1.14 ± 0.04 | <0.001* | 0.57 | (0.68 - 0.92) |
| | Mhd2 | All stages | 0.80 ± 0.06 | -1.02 ± 0.04 | <0.001* | 0.61 | (0.69 - 0.91) |
| Timings | TMhd1 | All stages | 0.01 ± 0.00 | 0.30 ± 0.00 | <0.001* | 0.12 | (0.01 - 0.02) |
| | TMhd2 | All stages | 0.01 ± 0.00 | 0.31 ± 0.00 | <0.001* | 0.11 | (0.00 - 0.01) |
| Speeds | MSGO | All stages | 0.76 ± 0.06 | 1.09 ± 0.05 | <0.001* | 0.53 | (0.64 - 0.89) |
| | MShd1down | All stages | 0.68 ± 0.07 | 0.83 ± 0.05 | <0.001* | 0.39 | (0.53 - 0.82) |
| | MShd2down | All stages | 0.59 ± 0.07 | 0.98 ± 0.05 | <0.001* | 0.33 | (0.45 - 0.73) |
| Acceleration | MAhd2down | All stages | 0.49 ± 0.10 | 3.34 ± 0.07 | <0.001* | 0.1 | (0.29 - 0.69) |
| ➢ Variables with shift between immature versus adult individuals | | | | | | | |
| Speeds | MSGC | immature | 1.01 ± 0.11 | 0.87 ± 0. 07 | <0.001* | 0.45 | (0.80 - 1.22) |
| | | adult | 2.88 ± 0.53 | -1.38 ± 0.59 | <0.001* | 0.59 | (1.78 - 3.97) |
| | MShd1up | immature | 0.72 ± 0.12 | 0.45 ± 0.08 | <0.001* | 0.23 | (0.48 - 0.97) |
| | | adult | 2.78 ± 1.14 | -1.91 ± 1.27 | 0.024 | 0.22 | (0.41 - 5.16) |
| Acceleration | MAGO | larvae | 0.74 ± 0.16 | 3.45 ± 0.1 | <0.001* | 0.18 | (0.42 - 1.06) |
| | | juvenile | 0.71 ± 1.66 | 3.50 ± 1.05 | 0.678 | 0.01 | (-2.84 - 4.25) |
| | | immature | 0.75 ± 0.16 | 3.45 ± 0.10 | <0.001* | 0.16 | (0.42 - 1.07) |
| | | adult | 4.07 ± 1.17 | -0.38 ± 1.30 | 0.002* | 0.37 | (1.64 - 6.50) |
| | MAhd1down | immature | 0.55 ± 0.15 | 3.25 ± 0.09 | <0.001* | 0.10 | (0.25 - 0.84) |
| | | adult | 3.79 ± 0.94 | -0.38 ± 1.05 | <0.001* | 0.43 | (1.82 - 5.75) |
| | MAGC | immature | 1.17 ± 0.17 | 3.09 ± 0.11 | <0.001* | 0.30 | (0.84 - 1.51) |
| | | adult | 5.08 ± 1.10 | -1.74 ± 1.22 | <0.001* | 0.51 | (2.81 - 7.36) |
| ➢ Variable with differences between all developmental stages | | | | | | | |
| Speed | MShd2up | Larvae | 0.89 ± 0.11 | 0.39 ± 0.07 | <0.001* | 0.39 | (0.66 - 1.11) |
| | | Juveniles | -0.76 ±0.73 | 1.55 ± 0.46 | 0.314 | 0.07 | (-2.31 - 0.79) |
| | | Adults | 3.96 ± 0.86 | -3.16 ± 0.96 | <0.001* | 050 | (2.17 - 5.76) |

** Denotes a significant difference*
*Same abbreviations as for table 1.*

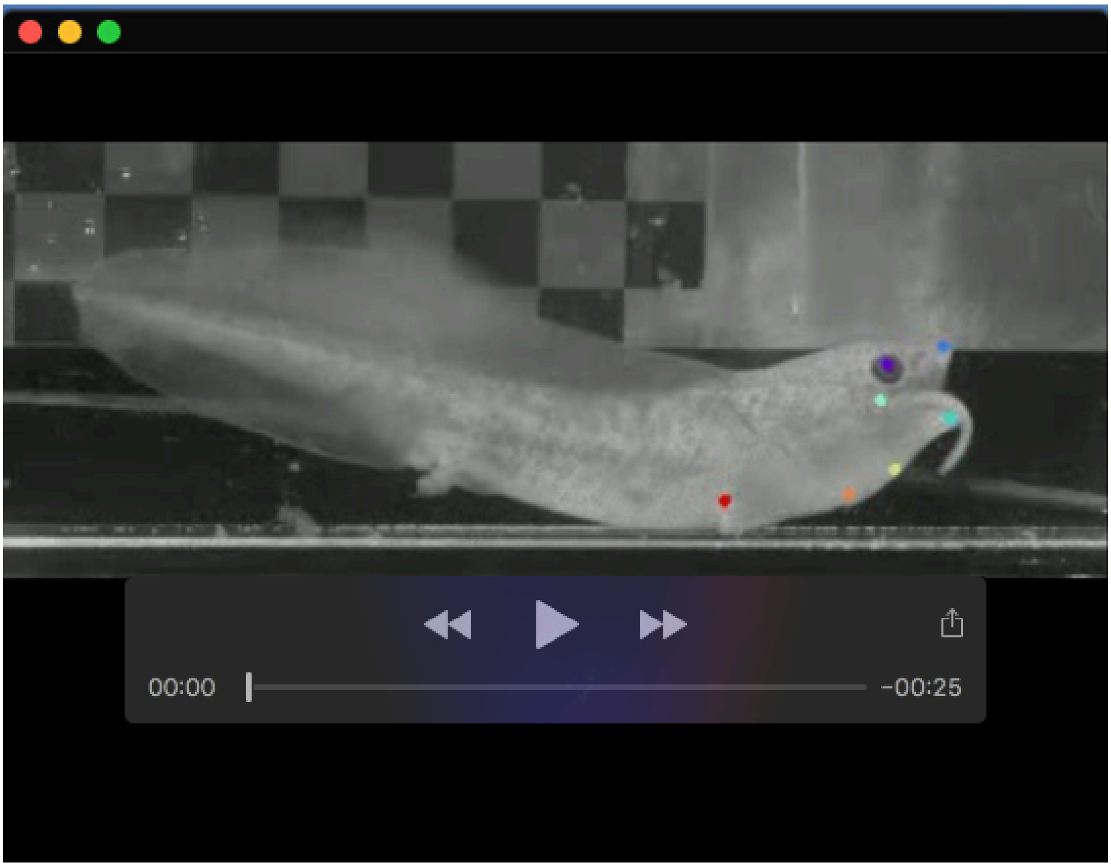


**Movie 1. Typical suction feeding at larval stage 54.**

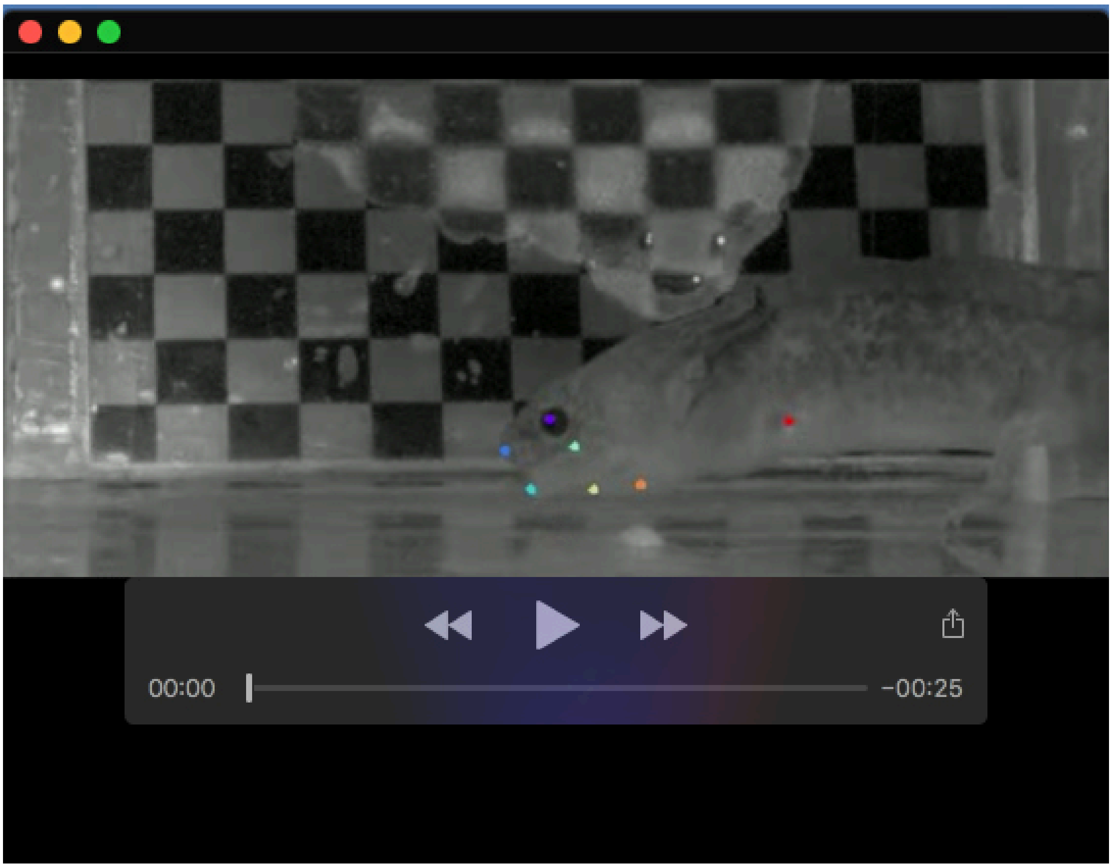


**Movie 2. Typical suction feeding at juvenile stage.**

Biology Open • Supplementary information

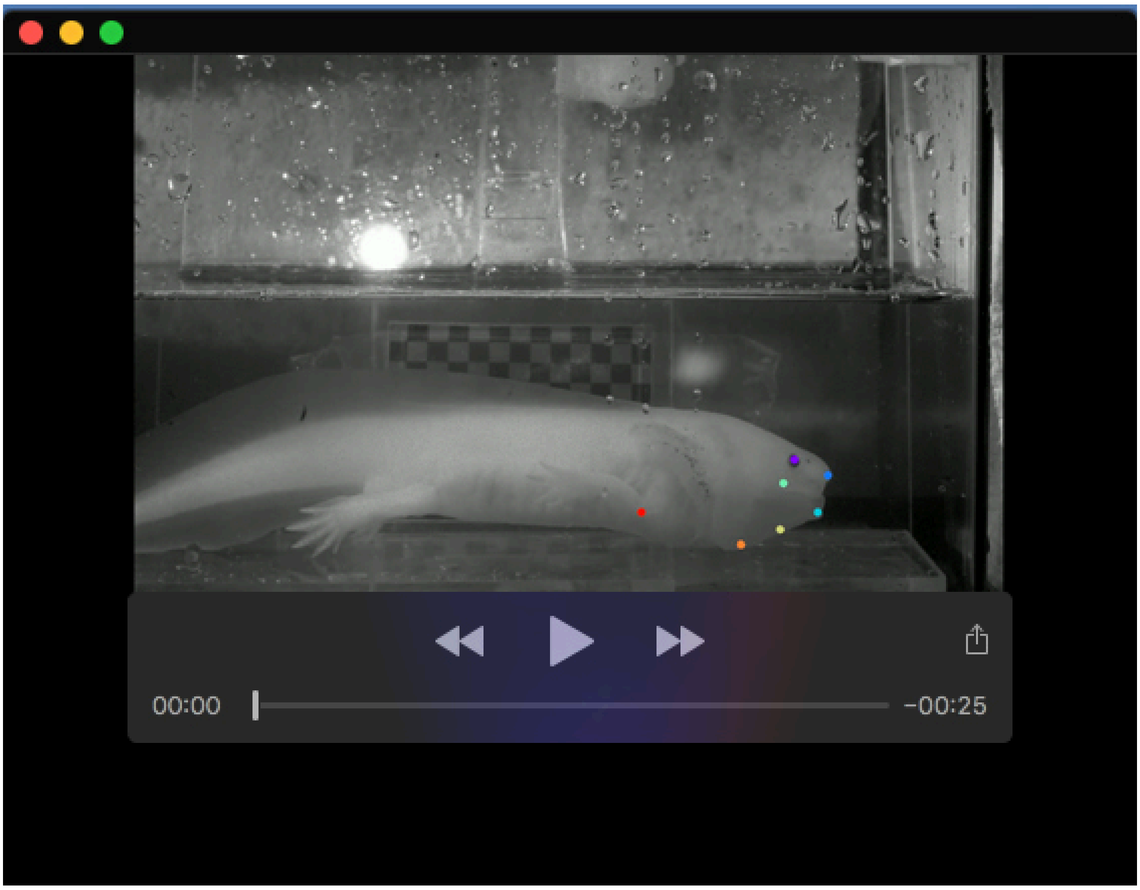


**Movie 3. Typical suction feeding at adult stage.**

Biology Open • Supplementary information
